# Development of a Broadly Protective, Self-Adjuvanting Subunit Vaccine to Prevent Infections by *Pseudomonas aeruginosa*

**DOI:** 10.3389/fimmu.2020.583008

**Published:** 2020-11-17

**Authors:** Sayan Das, Debaki R. Howlader, Qi Zheng, Siva Sai Kumar Ratnakaram, Sean K. Whittier, Ti Lu, Johnathan D. Keith, William D. Picking, Susan E. Birket, Wendy L. Picking

**Affiliations:** ^1^Department of Pharmaceutical Chemistry, School of Pharmacy, University of Kansas, Lawrence, KS, United States; ^2^Hafion LLC, Lawrence, KS, United States; ^3^Department of Medicine and Gregory Fleming James Cystic Fibrosis Research Center, School of Medicine, University of Alabama at Birmingham, Birmingham, AL, United States

**Keywords:** *Pseudomonas aeruginosa*, type III secretion system, vaccine, IL-17, opsonophagocytosis, protective efficacy, PcrV, PopB

## Abstract

Infections caused by the opportunistic pathogen *Pseudomonas aeruginosa* can be difficult to treat due to innate and acquired antibiotic resistance and this is exacerbated by the emergence of multi-drug resistant strains. Unfortunately, no licensed vaccine yet exists to prevent *Pseudomonas* infections. Here we describe a novel subunit vaccine that targets the *P. aeruginosa* type III secretion system (T3SS). This vaccine is based on the novel antigen PaF (Pa Fusion), a fusion of the T3SS needle tip protein, PcrV, and the first of two translocator proteins, PopB. Additionally, PaF is made self-adjuvanting by the N-terminal fusion of the A1 subunit of the mucosal adjuvant double-mutant heat-labile enterotoxin (dmLT). Here we show that this triple fusion, designated L-PaF, can activate dendritic cells *in vitro* and elicits strong IgG and IgA titers in mice when administered intranasally. This self-adjuvanting vaccine expedites the clearance of *P. aeruginosa* from the lungs of challenged mice while stimulating host expression of IL-17A, which may be important for generating a protective immune response in humans. L-PaF’s protective capacity was recapitulated in a rat pneumonia model, further supporting the efficacy of this novel fusion vaccine.

## Introduction

*Pseudomonas aeruginosa* (Pa) (a table of acronyms is provided in the Supplementary Information) is an important opportunistic human pathogen that causes severe infections in patients with burns, severe wounds, pneumonia, and critically-ill patients who require intubation (ventilator-associated pneumonia) or catheterization (urinary tract infections) (1, 2). Treating Pa infections has become increasingly difficult due to antibiotic resistance (1) and multidrug-resistant (MDR) Pa is classified as a serious threat in the CDC Antibiotic Resistance Threats report in 2019 (3). The report states that in 2017 there were ~32,600 cases of MDR Pa infections in hospitalized patients causing an estimated 2700 deaths and costing $757 million in health care costs in the US. In the military, Pa was reported in 2011 to be responsible for a quarter of the 6% of patients with infection-related billing in Iraq and Afghanistan (4). A 2016 report describes Pa as the most common Gram-negative infection among troops with combat-related injuries in Afghanistan with 10% being MDR (4). Pa is also a serious cause of pulmonary infections in cystic fibrosis (CF) patients with >70% of this group being chronically colonized by their teens (2). These Pa pulmonary infections decrease lung function, thereby priming the patient for other opportunistic infections and contributing to a shortened life span in CF patients. Although certain diseases and occupations increase the risk of MDR Pa infection, aging is perhaps the biggest risk factor for acquiring lethal Pa infection (4).

Perhaps the greatest public health achievement of our time is vaccination, however, there are no licensed prophylactic vaccines to prevent Pa infections. Vaccines that are currently being developed can largely be separated into distinct groups: 1) whole killed or live-attenuated; 2) outer membrane vesicles (OMV)/LPS; 3) specific surface proteins of the flagella, pili or the type III secretion system (T3SS); 4) or a combination of these (4, 5). While the first group could give the best protective immune response (6–8), it suffers from potential reactogenicity and risks when administrated to immunocompromised individuals (9). The second group including OMVs would produce a serotype-specific response with the outer membrane proteins being either difficult to purify or prepared as only the surface exposed portion of the antigen (4, 10–12). The third group (specific surface proteins) continues to include viable vaccine candidates since these surface exposed proteins are often responsible for pathogenesis and do not possess the reactogenicity or serotype specificity of the other candidates (4, 5). The proteins of the type III secretion system (T3SS) and exposed parts of the T3SS apparatus (T3SA) are examples of this group. Proteins here include PcrV, the T3SA tip protein for Pa, and PopB, the first of two T3SS translocator proteins. The use of PcrV and PopB (with its cognate chaperone PcrH) as protective antigens has been shown to provide a level of protection in mice within the context of other adjuvants such as CpG, curdlan and alum (13–17).

Vaccines based on the *Shigella* T3SS tip (IpaD) and the first translocator (IpaB) proteins have been shown to be broadly protective against *Shigella* spp. infections (18–21). The success of the *Shigella* T3SS antigens can be attributed, in large part, to the newly available double-mutant labile toxin (dmLT) from Enterotoxigenic *Escherichia coli* (22). dmLT has been demonstrated to elicit a balanced Th1/Th2 immune response, along with IL-17, but more importantly, it is a strong stimulator of the mucosal response required for protection against pathogens such as *Shigella* spp., *S. enterica* and, potentially, Pa. Unfortunately, dmLT has been associated with an increased risk of facial paralysis in vaccine recipients when administered intranasally (10). However, LTA1, the active moiety of dmLT, retains the toxin’s ADP-ribosylation (ADPr) activity and the ability to promote dendritic cell (DC) maturation (23), but does not possess the B subunit responsible for targeting it to neuronal gangliosides (23). LTA1 also stimulates a balanced Th1/Th2 response along with a mucosal response characterized by production of mucosal IgA and IL-17 (23). Recently, it was shown that the addition of LTA1 to Fluzone^®^ increased IgA while decreasing levels of IL-6 post-H1N1 challenge (24).

Because any vaccine designed to prevent shigellosis must be economical for use in low- and middle-income countries, IpaD and IpaB were genetically fused to form DBF (25). DBF was as protective as IpaD and IpaB when delivered intranasally (25, 26). Likewise, fusion proteins consisting of the *Salmonella enterica* T3SS tip and translocator proteins have been found to be attractive vaccine candidates (27). It is important to note that these antigens from *Shigella* and *S. enterica* elicited serotype-independent protection against their respective parent pathogens. Such protection is unique in that the majority of the potential vaccines against these pathogens target surface LPS, thereby rendering them serotype-specific. In keeping with the need for an economical vaccine, the risks of dmLT and success of LTA1, we genetically fused LTA1 to DBF to form L-DBF. When delivered intranasally, L-DBF is as protective as IpaD+IpaB+dmLT or DBF+dmLT (manuscript in preparation). In addition to requiring a single bioreactor run, L-DBF will not require parallel dose escalation studies of the antigen and adjuvant since L-DBF is self-adjuvanting. Furthermore, unlike the dmLT+DBF formulation, the entire L-DBF (adjuvant and antigen) can be taken up by a single dendritic cell, which should enhance and better target the immune response.

The equivalents of IpaD and IpaB from the *Shigella* T3SS are PcrV and PopB, respectively, in *P. aeruginosa* (28) with amino acid sequences being highly conserved among Pa serotypes (29)(https://pseudomonas.com/). Therefore, PcrV and PopB should provide serotype-independent protection as was seen in *Shigella* spp. and *S. enterica*. Rather than chose between PcrV or PopB or use them together with dmLT, these antigens were genetically fused to form PaF (Pa-Fusion) and LTA1 genetically fused to form a single fusion protein, L-PaF (LTA1-PcrV-PopB), as was performed with L-DBF. Not only does L-PaF eliminate the need for dmLT, but it also allows for simultaneous uptake of adjuvant-antigen by antigen presenting cells to enhance cellular immunity. Additionally, it simplifies formulation and reduces the cost of production, both concepts that are important to consider early in the effort to produce a viable vaccine. The goal of this study is to test the hypothesis that the single fusion protein, L-PaF, when delivered intranasally, can protect mice against Pa, and elicit a corresponding immune response that induces robust IL-17 levels and high antibody titers that mediate opsonophagocytosis of Pa. Both of these responses are postulated to be required of a successful Pa vaccine candidate (4). Here we present the proof of concept that L-PaF is a potential vaccine candidate that is superior to the single Pa proteins.

## Materials and Methods

### Materials

pET and pACYCDuet-1 plasmids, ligation mix and competent *E. coli* were from EMD Millipore (Billerica, MA). Restriction endonucleases were from New England Biolabs (Ipswich, MA). Chromatography columns were from GE Healthcare (Piscataway, NJ). All other reagents were from Sigma or Fisher Scientific and were chemical grade or higher. Animals were infected with Pa strains mPA-0831 or PA15808959. Both strains are clinical isolates cultured from sputum samples from patients with cystic fibrosis at University of Alabama at Birmingham (under IRB #160720008). mPA-0831 was characterized as a mucoid phenotype. PA15808959 was classified as a non-mucoid phenotype. Pa14 and Pa14 *pscD* were from Dara Frank (Medical College of Wisconsin).

### Protein Production

The amino acid sequences are provided in the Supplementary Information. The plasmid used for expression of full-length PcrV has been previously described (30). To produce full-length PopB, the *popB* gene was synthesized and cloned into the second multiple cloning site in pACYCDuet-1 while the *pcrH* gene, the PopB chaperone, was synthesized and cloned into the first multiple cloning site. The resulting plasmid (*popB*/*pcrH*/pACYCDuet-1) was used to transform *E. coli* Tuner(DE3) for co-expression. To produce PaF, *pcrV* was cloned in-frame 5’ to *popB* in the *popB/pcrH*//pACYCDuet-1 plasmid. The resulting plasmid (*pcrV-popB/pcrH*//pACYCDuet-1) was used to transform *E. coli* Tuner(DE3) for co-expression. To produce LTA1-GSAAS-PaF (L-PaF), *eltA1* (the gene for full-length LTA1) was cloned in-frame 5’ with the linker gggtccgcggcatcc to *pcrV* in the PcrV-PopB+PcrH/pACYCDuet-1 plasmid. The resulting plasmid (*eltA1-pcrV-popB/pcrH*//pACYCDuet-1) was used to transform *E. coli* Tuner (DE3) for co-expression.

PcrV was purified as previously described (30) with the addition of a Q FF anion exchange chromatography (Q) column. The immobilized metal affinity chromatography (IMAC) purified His-Tag (HT) PcrV was loaded onto a Q column in 50 mM Tris-HCl pH 8.0 and eluted with a gradient of 50 mM Tris-HCl pH 8.0 containing 1M NaCl. The purified HT-PcrV was dialyzed in 10 mM sodium phosphate pH 7.2 with 150 mM NaCl (PBS) and stored at −80°C.

PopB, PaF, and L-PaF were purified in a manner similar to that of IpaB, SipB, YopB as previously reported (18, 31–33). Briefly, PopB, PaF, and L-PaF were each co-expressed with HT-PcrH, which forms a stable, soluble complex. PopB/HT-PcrH was grown shake-flasks in Terrific Broth (TB) containing 25 µg/ml chloramphenicol until the A_600_ reached 0.8 at which point protein expression was induced with 1 mM IPTG. After 3 h, bacteria were collected by centrifugation, resuspended in IMAC binding buffer (20 mM Tris pH 8.0, 500 mM NaCl, 10 mM imidazole), lysed and the suspension clarified by centrifugation. The supernatant containing the PopB/HT-PcrH complex was loaded onto a 5 ml IMAC cartridge. After elution of the complex with IMAC elution buffer (20 mM Tris pH 8.0, 500 mM NaCl, 500 mM imidazole), the complex was dialyzed into 50 mM Tris pH 8.0 and loaded onto a Q column. The complex was eluted using a gradient of 50 mM Tris pH 8 containing 1M NaCl. Lauryldimethylamine oxide (LDAO) was added to a final concentration of 0.1% to release the HT-PcrH. When the LDAO-treated PopB/HT-PcrH complex was passed over an IMAC column, the PopB was collected in the flow-through with the HT-PcrH being retained on the IMAC column. Finally, PopB was dialyzed into PBS with 0.05% LDAO and stored at −80°C. PaF was purified in the same manner.

For L-PaF, bacteria were grown in a fed-batch mode using a 10L bioreactor (Labfors 5, Infors USA Inc., MD) equipped with polarographic dissolved oxygen probe (pO_2_), pH probe (Hamilton Company) and advanced fermentation software. Materials were prepared as per the manufacturer’s specifications (http://www.infors-ht.com.cn/uploadfile/2018/0515/INFORS-HT_cookbook_en.pdf). Briefly, pre-culture was prepared by inoculating a 25-μl aliquot of frozen glycerol stock into 50 ml of TB supplemented with chloramphenicol (34 µg/ml) and allowed to grow overnight at 30°C with shaking at 200 rpm. The inoculum was made by transferring cells from the pre-culture to 1 L of TB medium with the same antibiotic and grown at 30°C until reaching an A_600_ of ∼2.0. In the next step, ~800 ml of inoculum was transferred to the sterilized bioreactor containing 9 L of the TB medium with appropriate antibiotic. The culture temperature was maintained at 30°C and with a pH of 7. Other parameters such as stirrer speed, gas mix, and flow were set in cascade mode as a function of pO_2_ (30%). Protein expression was induced by addition of IPTG to 1 mM when the culture reached an A_600_ of ∼25. After 3 h, bacteria were collected by centrifugation, washed and resuspended in IMAC binding buffer (20 mM Tris pH 7.9, 500 mM NaCl, 10 mM imidazole) with 0.1 mM AEBSF Protease Inhibitor and lysed using a microfluidizer at 18,000 psi with three passes. The cellular debris was removed by centrifugation at 10,000xg for 30 min and loaded onto an IMAC column. The remainder of the purification was as for PopB with 20% glycerol included in the buffers of the final IMAC column. LPS levels were determined using a NexGen PTS with EndoSafe cartridges (Charles River Laboratories, Wilmington, MA). All proteins had LPS levels <5 Endotoxin units/mg. The purity of the proteins was assessed by standard SDS-PAGE (**Supplementary Figure S1A**).

### ADP-Ribosylation Assay

LTA1 from lethal toxin of Enterotoxigenic *E. coli* possesses ADP-ribosylation (ADPr) activity, which is required for adjuvanticity (22). The activity assay was carried out in a 15 µl reaction mix containing 50 mM Tris-HCl pH 7.5, 1 mM EDTA, 1 mM DTT, 1.7 µM ARF4 substrate, 8.3 µM biotin-labeled NAD^+^ (BPS Biosciences, San Diego, CA) and 1.7 µM LTA1-PaF. After 1 h incubation at 37°C, the reaction was stopped by adding 5 µl of 3X SDS-PAGE sample buffer and boiling for 5 min. Proteins were separated by 15% SDS-PAGE, subjected to western blot analysis with the biotin moiety on the ribosylated proteins visualized with IR dye 800 CW Streptavidin (Li-Cor, Lincoln, NE).

### Bone Marrow-Derived Dendritic Cell Assay

The animal studies were reviewed and approved by the University of Kansas Institutional Animal Care and Use Committee practices (protocol AUS 221-03). Bone marrow dendritic cells (BMDC) were isolated as previously described (34). Briefly, BALB/c mice (Charles River Laboratories, Wilmington, MA) were euthanized and femurs were extracted. The ends of the femurs were cut off and the bone marrow flushed with cold media. After harvesting the cells by centrifugation, they were incubated with RBC lysis buffer (Sigma), neutralized with complete RPMI and filtered with a 70 µm filter. The resulting single cell suspension was grown in presence of granulocyte-macrophage colony-stimulating factor (GM-CSF) (Invitrogen) at a concentration of 20 ng/ml of complete RPMI. On days 3 and 6, 50% of the media was replaced with fresh complete RPMI containing 20 ng/ml of GM-CSF. On day 8, loosely adherent cells (BMDCs) were harvested. The harvested cells were seeded into 6 well plates (10^6^ cell/well) and treated with recombinant proteins at a low, medium and high antigen dose. After 32 h, cells were harvested, blocked and stained with fluorochrome tagged antibodies. The cells were then analyzed by flow cytometry.

### Mice and Immunizations

Six to eight week old BALB/c mice (n=10) (Charles River Laboratories, Wilmington, MA) were used for all experiments. Prior to administration, the following were prepared in 30 µl volumes: PcrV (10 µg), PopB (2.5 µg), PcrV+PopB (10 µg+2.5 µg), or PaF (20 µg) and all were admixed with dmLT (2.5 µg). Additionally, in a 30 µl vaccination volume, three concentrations of L-PaF, 1, 10, 20 µg; and PBS were prepared for IN delivery. For immunizations, mice were anesthetized using isoflurane and vaccine formulations administered intranasally (IN) as previously described (18). Immunizations were on days 0, 14 and 28 for this study. Blood was collected prior to each vaccination and at days 42 and 55. In a second study, mice (n=10) were vaccinated as described with only PBS, PaF+dmLT, and 1, 10, or 20 µg of L-PaF

### Antigen-Specific IgG and IgA

Antibodies specific for PcrV and PopB were determined by ELISA, as described previously with minor modifications (18). Briefly, 96-well plates coated with PcrV or PopB (1 µg/ml in PBS) were blocked overnight with 10% milk in PBS. Each well was incubated with serum samples for 1 h at 37°C. After washing the plates with PBS-Tween (0.05%), secondary antibody (KPL, Gaithesburg, MD) was added and incubated for 1 h at 37°C. Levels of IgG (H+L) and IgA were determined with respective horseradish peroxidase-conjugated secondary antibodies (human serum adsorbed) raised in goat (Southern Biotech, Birmingham, AL). 3,3′,5,5′-Tetramethylbenzidine (TMB) substrate was added and reaction was stopped with H_3_PO_4_. Endpoint titers were calculated and represented as ELISA units per ml (EU ml^-1^).

### Opsonophagocytosis Assay

Opsonophagocytic killing (OPK) was carried out as previously described (35). Briefly, Pa strain mPA08-31 or PA15808959 was grown overnight. A new culture was started by adding 100 µl of the overnight culture to 5 ml of LB media and grown to A_600_ of 0.3. Bacteria were collected by centrifugation and a portion of the resuspension adjusted to a concentration of 2x10^7^ cells/ml in Minimal Essential Medium (MEM, ThermoFisher, Waltham, MA) containing 10% bovine serum albumin (BSA, Sigma, St. Louis, MO). The J774A.1 (ATCC, Manassas, VA) macrophage cell line was grown to 90% confluency in Dulbecco’s Modified Eagle’s Medium (DMEM, ThermoFisher, Waltham, MA) and was adjusted to allow for a final multiplicity of infection (MOI) of 0.1 in 10% MEM-BSA. At 42 days post-immunization (DPI) sera from the vaccinated mouse groups (5 µl from each mouse were mixed) were heat treated at 56°C to destroy complement. Serum from mice vaccinated with 5x10^6^ boiled clinical Pa isolate mPa 08-31 (whole cell killed - WCK) was used as a positive control for the mPA08-31 assay. Serum (1:500), bacteria and macrophages were mixed at a 1:1:1 ratio to a final volume of 300 µl and kept for 30 min at 37°C. The suspension was then serially diluted and plated on *Pseudomonas* Isolation Agar (PIA, BD, Franklin Lakes, NJ). Percent killing = [((CFU from T_0_) – (CFU from T_30min_))/(CFU fromT_0_) × 100]. An appropriate serum control was used where MEM-BSA was used in place of serum in the published protocol. Technical quadrulets from each group were assessed and statistical comparisons made as described below. Pa14 and Pa14 *pscD*, a T3SS null, were grown in the same method as mPA08-31. Day 42 post-vaccination rat sera were assessed for OPK by the same methodology.

### Cytotoxicity Assay

A549 cells were seeded in 96 well plates (10^4^ cells/well) and allowed to attach overnight. Bacterial strains (PA15808959) were grown to log phase and diluted to obtain the desired MOI (1:10). The serum was heat inactivated at 56°C for 30 min and incubated with the bacteria for 15 min at room temperature at a dilution of 1:500. A549 cells were infected with the bacteria incubated with various serum or without serum (control). The plates were incubated at 37°C, 5% C0_2_. After 3 h, the plates were centrifuged at 500 x g for 5 min and 50 µl of the supernatant was transferred to a 96 well plate. 50 µl of the CytoTox 96 reagent (Promega, Madison, WI) was added to each well and incubated at room temperature for 15 min. 50 µl of the stop solution was added and the plate was read at 490 nm. The percent cytotoxicity was calculated using the formula: (Experimental-ES-TS+M)/(T_max_-TS); where Experimental is OD from the well being tested, ES is OD from bacteria only, TS is OD from untreated cells, M is OD from media and T_max_ is OD from lysed cells (obtained by adding Lysis solution 45 min prior to end of incubation).

### Pseudomonas aeruginosa Challenge

Pa strain mPA08-31 was streaked onto *Pseudomonas* isolation agar (PIA) and incubated overnight at 37°C. Several isolated colonies were used to inoculate 50 ml LB and grown at 37°C with 250 rpm shaking the A_600 nm_ reached ~0.3. The Pa were collected by centrifugation, resuspended in PBS and diluted to 2.5x10^7^ CFU/30 µl. On day 56, mice were anesthetized by isoflurane and challenged IN. On day 3 post-infection, mice (n=5) from each group were euthanized and the lungs, liver, spleen and bone marrow were collected and processed to assess the immune response including quantifying antigen-specific IFN-γ and IL-17A secreting cells, and secreted cytokine levels. Additionally, the CFU/lung was assessed for each mouse by plating a portion of the lung extract on PIA. On day 4 post-infection, the remaining five mice were euthanized and the lungs processed to determine CFU/lung with no immune response assessed.

As a second challenge, mice (n=10) that had been vaccinated with PBS, PaF+dmLT, and 1, 10, or 20 µg of L-PaF were challenged on day 56 with 1x10^7^ CFU/30 µl of the Pa strain PA15808959. A lower challenge dose and earlier euthanasia were required since this strain is more virulent than mPA08-31. Mice (n=5 on each day) were euthanized for assessment of the immune response and CFU/lung burden on days 2 and 3.

### IFN-γ and IL-17 ELISpot

Mouse cells isolated from spleens and lungs were incubated for 48 h at 37°C in the presence of 5 µg/ml PcrV or PopB, in plates coated with antibodies against IFN-γ or IL-17A for a dual-color assay as per manufacture’s specifications (Cellular Technology Limited). The cytokine secreting cells were quantified using a CTL immunospot reader.

### Cytokine Determinations

Splenocytes were incubated with 10 µg/ml PcrV, PopB or PBS for 48 h at 37°C. Supernatants were collected and analyzed with U-PLEX kits for cytokines: IFN-γ, IL-10, IL-12p70, IL-17A, IL-1β, IL-2, IL-4, IL-5, IL-6, and TNF-α. Cytokine concentrations were determined using an MSD plate reader with associated analytical software (Meso Scale Discovery, Rockville, MD). While multiple cytokines were measured, the two that are focused on in this report are IL-17A and IFN-γ as others did not show significant changes.

### Analysis of Immune Markers Associated With Protection

Immune markers associated with protection from *Pseudomonas aeruginosa* were identified by comparing immunological data (including cytokine levels, cytokine secreting cells, and antibody secreting cells) from unchallenged mice euthanized 56 days after vaccination to the group average CFUs per lung of challenged mice two days after challenge. The correlation between immune markers and group average lung CFUs was determined using the Pearson correlation coefficient, with significant correlations having a two-tailed p-value less than 0.05.

### Rat Studies

The rat animal protocols were reviewed and approved by the University of Alabama at Birmingham Institutional Animal Care and Use Committee Practices (protocol AUS IACUC-21441). Sprague-Dawley rats (bred at the University of Alabama at Birmingham) were immunized at 8 weeks of age (Day 0) and subsequently at days 14 and 28. Blood was collected *via* retro-orbital bleed at days 0, 14, 28, 42, and 56 and processed for serum. Rats were anesthetized by isoflurane and vaccinated by the IN route with PBS, 50 or 100 µg of L-PaF in a volume of 50 µl. Pa strain mPA08-31 was grown in LB overnight to an OD A_600_ of 1.0. Bacteria were collected and were collected by centrifugation, resuspended in PBS and diluted to 1x10^7^ Pa/100 µl. On day 56, rats were anesthetized by isoflurane and challenged *via* intranasal instillation. On day 3 post-infection, rats were euthanized *via* pentobarbital injection and the CFU/lung was assessed on PIA plates.

### Statistical Analysis

GraphPad Prism 8.1.2, Python and R were used to generate graphics and to perform all statistical comparisons. Differences among treatment groups were analyzed using ANOVA. Challenge groups were compared to PBS with Dunnett’s multiple comparisons tests. A *p* value of less than 0.05 was considered significant for all comparisons. * P< 0.05; ** P<0.01; *** P< 0.001.

## Results and Discussion

### *In Vitro* Characterization of L-PaF

In this study, PopB, PaF, and L-PaF were purified based on our knowledge of translocator protein behavior (**Supplementary Figure S1A**). These proteins were co-expressed with the HT-PcrH chaperone as has been described for PopB by others (17). Stable T3SS translocator expression requires co-expression with its cognate chaperone to produce a soluble translocator:chaperone complex (36). The PopB-HT-PcrH complexes were then dissociated using the detergent LDAO, resulting in stable PopB-containing protein preparations as previously described for *Shigella*, *Salmonella enterica* and *Yersinia* (18, 25, 26, 31, 37). While others have shown that IN administration with PopB/PcrH can protect mice against Pa challenge with PcrH appearing to not contribute to the protective efficacy (17), the translocator:chaperone complex from *Shigella* (IpaB/IpgC) was partially protective when delivered orally using the mouse model (21). However, no protection was seen when a guinea pig keratoconjunctivitis *Shigella* infection model was used after IN delivery. In contrast, IpaB without its chaperone was protective in the guinea pig model after IN delivery (E. Barry, W.L.Picking, unpublished results). Thus, the PopB, PaF and L-PaF used in this study do not contain PcrH.

To be a viable self-adjuvanting vaccine, the LTA1 of L-PaF must retain its ADPr activity. Thus, an ADPr assay was performed (**Supplementary Figure S1B**). Biotinylated-NAD^+^ was incubated with L-PaF and ARF4. The resulting blot was probed using IR-labeled streptavidin to reveal that the biotinylated-ADPr was transferred to ARF4 by the L-PaF. This is not confirmation that the LTA1 retains adjuvanticity, however, the absence of such activity would have deemed the L-PaF non-viable as a self-adjuvanting vaccine candidate. It should be noted that recombinant LTA1 sequesters to *E. coli* inclusion bodies and must be refolded after solubilization in a denaturing agent such as urea, which increases difficulties in formulation. Therefore, it was not added as a comparator in these studies.

**Figure 1:**
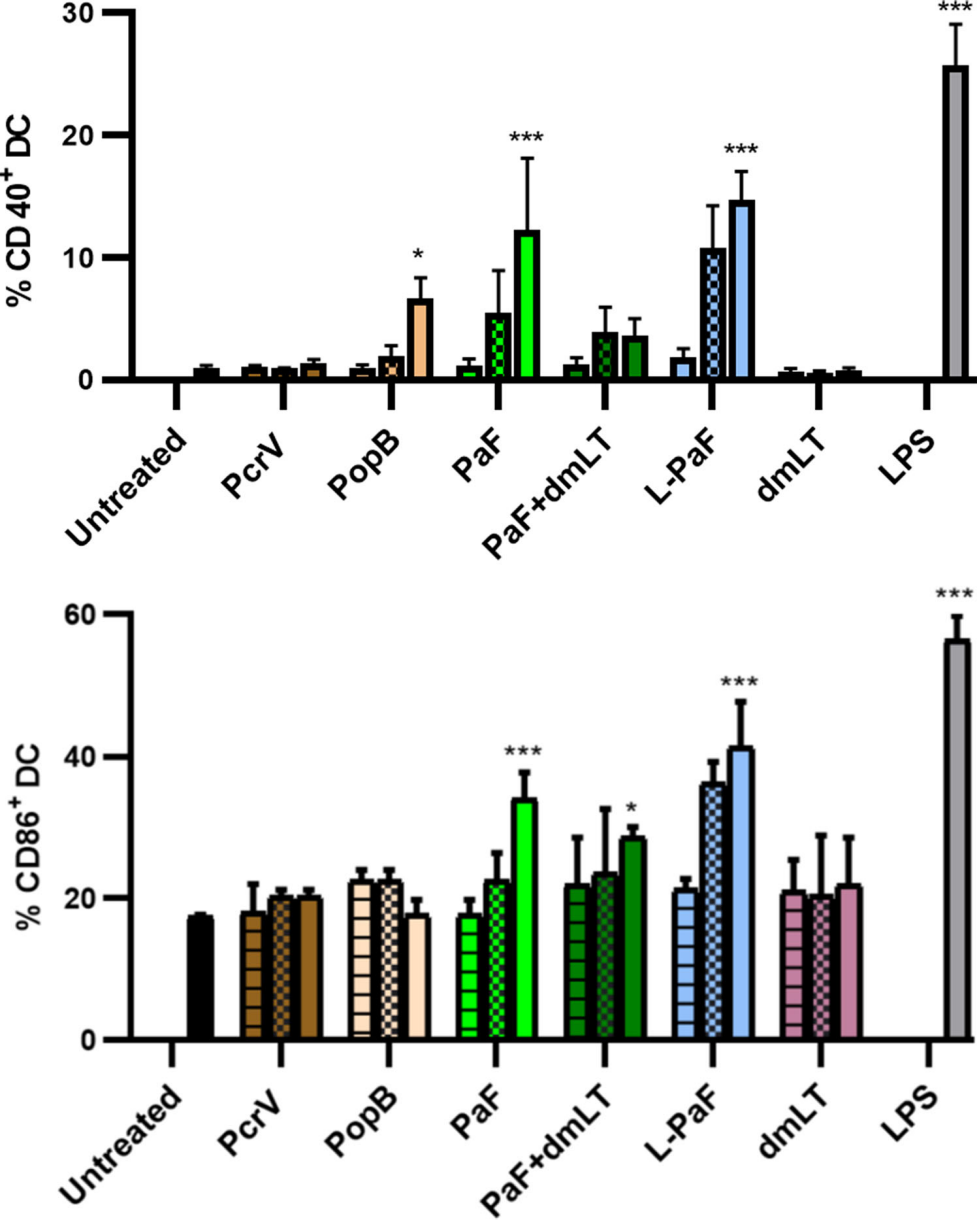
Assessment of LTA1-GSAAS-PaF (L-PaF) effects on Bone marrow dendritic cell (BMDC) maturation. Mouse BMDCs were untreated or treated for 32 h as indicated with low (horizontal stripes), medium (checkerboard), or high (solid) concentrations of the indicated proteins. For L-PaF the protein concentrations used were 1.0, 0.1, and 0.01 µg/ml. To ensure the same molar amount of antigen present, for PaF alone, we used 0.77, 0.07, and 0.007 µg/ml. Approximately half of the latter amounts were used for stimulation with PcrV and PopB alone to give equivalent molar concentrations. *Salmonella enterica* Typhimurium LPS (made by Westphal method in the Picking lab) was used at 10 µg/ml. Co-staining of CD11c (DC marker) with the maturation markers CD40^+^ (top), CD86^+^ (bottom) was determined by flow cytometry as a measure of dendritic cell maturation. Results are represented as average of triplicates with these results being representative of five independent experiments. Significance was calculated by comparing to untreated groups using two-way ANOVA. *p < 0.05; ***p < 0.001.

In addition to the ADPr activity, LTA1 should possess the ability to induce BMDC maturation as a sign that it retains its adjuvant activity (23). Thus, BMDCs were either untreated, treated with LPS, or treated with three increasing concentrations of PcrV, PopB, PaF, PaF+dmLT or L-PaF (34). After selecting for CD11c^+^ cells (DCs), the percentage of CD40^+^ or CD86^+^ was quantified using flow cytometry (**Figure 1**). These two markers are important since an elevated level of CD86^+^ cells is an indication of co-stimulation corresponding to increased numbers of CD28^+^-positive lymphocytes (T-cells) and cytokine induced clonal expansion while increases in CD40^+^ levels may be an indicator of the mechanism of IL-17 induction elicited by L-PaF (38). Only the highest PopB concentration elicited a significant percent of CD40^+^ cells with no significant increase in CD86^+^ cells. PcrV did not elicit increases in either population. This was expected with PcrV and PopB being included as a baseline for PaF and L-PaF. Interestingly, the highest PaF concentration elicited significant increases in CD40^+^ and CD86^+^ populations while the highest PaF+dmLT concentration elicited a significant percentage in only the CD86^+^ cells. It is unclear why the dmLT or lower dmLT+PaF concentrations did not increase the CD40^+^or CD86^+^ percentages, but similar trends were seen with *Shigella* DBF groups (34). Nevertheless, PaF alone would not be considered a viable vaccine formulation in humans because it is a single, non-adjuvanted protein. Finally, as anticipated, L-PaF treatment induced an increase in the percent of both CD40^+^ or CD86^+^ cells in a dose dependent manner. Thus, BMDC maturation indicated that L-PaF could induce DC activation and maturation in the mice and it was thus moved forward toward into vaccination trials.

### L-PaF Elicits High Titers of Anti-PcrV and Anti-PopB IgG and IgA, Which Exhibit Pa killing and Inhibit Pa Cytotoxicity

**Figure 2:**
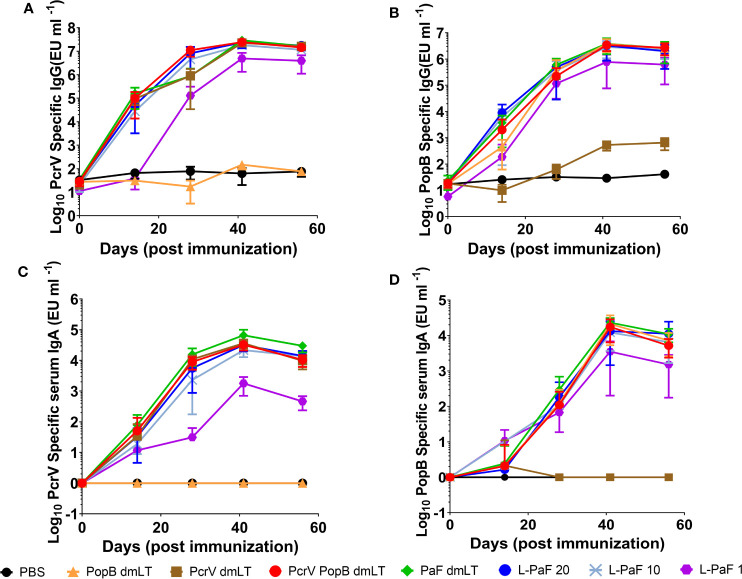
Kinetics of serum IgG and IgA responses. Mice were vaccinated IN three times (Day 0, 14, and 28). Blood samples were collected and serum titers for IgG **(A, B)** and IgA **(C, D)** specific for PcrV **(A, C)** or PopB **(B, D)** were measured by ELISA. The individual titers are represented as EU ml^-1^. Each point represents the mean and error bars represent SD of each group (n=10/group).

Having established that L-PaF can induce BMDC maturation *in vitro*, mice were vaccinated with L-PaF to determine if the LTA1 could elicit high antibody titers against PcrV and PopB. Based on previous work with *Shigella* proteins, mice were vaccinated intranasally (IN) with three concentrations of L-PaF, 1, 10, 20 µg, as well as PcrV (10 µg), PopB (2.5 µg), PcrV+PopB (10 µg+2.5 µg, respectfully), or PaF (20 µg) admixed with dmLT (2.5 µg). PBS was used as a negative control. Serum IgG and IgA titers against PcrV and PopB for these mice were determined by ELISA (**Figure 2**). Serum anti-PcrV IgG titers were comparable among all the groups regardless of the PcrV-containing formulation used, with the exception of the PopB alone and 1 µg L-PaF dose group (**Figure 2A**). While the anti-PcrV IgG final titer of this group was comparable to the other formulations, the kinetics of the anti-PcrV IgG showed a noticeable delay in the response. Similarly, the serum anti-PcrV IgA titers were comparable among the groups with exception of the PopB alone and 1 µg L-PaF group with the latter titers being delayed (**Figure 2C**). Unlike the anti-PcrV IgG, the anti-PcrV IgA titer for the 1 µg dose never reached the levels attained by the rest of the formulations. For PopB, the serum anti-PopB IgG kinetics were comparable among the groups of all formulations except for PcrV alone (**Figure 2B**). Serum anti-PopB IgA kinetics were also comparable in all groups of all formulations except for PcrV alone (**Figure 2D**).

**Figure 3:**
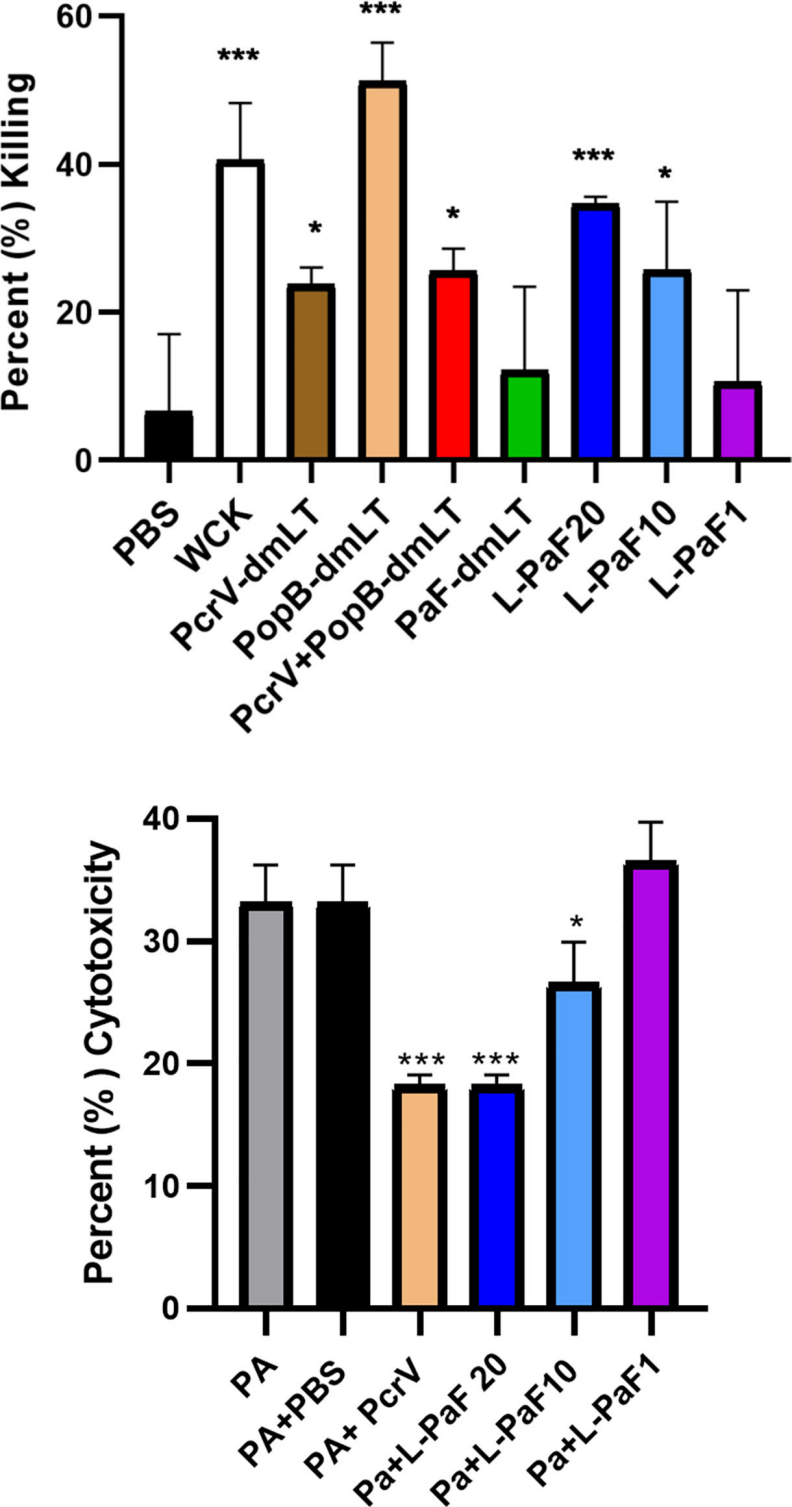
Functional activity of serum samples. Top [opsonophagocytic killing (OPK) activity]: Sera from each vaccinated mouse group (5 µl/mouse) were tested for their OPK activity after heat-inactivation of complement. The sera were combined with mPA08-31 and J774A.1 cells at an MOI of 0.1. WCK represent sera from mice vaccinated with the killed, whole-cell Pa strain mPA08-31. Killing was determined using (T_0_-T_30_)/T_0_x100. This is a representative experiment of quadruplicates for each sample. ***p < 0.005 and *p < 0.01 for respective groups were compared to PBS using a two-way ANOVA. **Bottom (Cytotoxicity inhibition):** A549 cells were seeded in 96 well plates (10^4^ cells/well) and incubated with serum treated PA15808959 at an MOI of 10 for 4 h. Cytotoxicity was calculated as described in Methods. Groups on x-axis are: PA, PA15808959 alone with no serum added; PA+PBS, PA15808959 with PBS vaccinated mouse serum; PA+PcrV, PA15808959 with PcrV vaccinated mouse serum; PA+L-PaF20, PA15808959 with 20 µg LTA1-GSAAS-PaF (L-PaF) vaccinated mouse serum; PA+L-PaF10, PA15808959 with 10 µg L-PaF vaccinated mouse serum; and PA+L-PaF1, PA15808959 with 1 µg L-PaF vaccinated mouse serum. Significance was calculated by comparing PA only with other groups using two-way ANOVA. *p < 0.05; **p < 0.01; ***p < 0.001.

To determine whether the antibodies elicited by L-PaF and antigens+dmLT could contribute to protection *via* bacterial killing, OPK assays were performed (**Figure 3****, top**). Sera from mice immunized with each formulation, as well as mice vaccinated with boiled mPA08-31 (WCK), were assayed for the ability to kill the Pa clinical isolate mPA08-31. WCK was used as a method to approximate maximal levels of OPK that could be obtained when LPS was included as an antigen. Sera from these mice exhibited ~40% OPK. While most of sera from antigen vaccinated groups demonstrated some level of OPK activity, the sera from mice vaccinated with 20 µg L-PaF were better at bacterial killing when compared to the antigens admixed with dmLT except in the case of PopB+dmLT. Furthermore, the OPK activity of the L-PaF was dose dependent. Although PopB exhibits higher OPK activity when compared to 20 µg L-PaF, the use of dmLT in the IN route is not deemed viable and PopB+dmLT requires the production of two proteins, which complicates formulation. The OPK activity of sera from the L-PaF vaccinated mice was not observed when a *pscD* null Pa mutant was used in the assay (data not shown). PscD is an essential T3SA structural component that is required for assembly of a functional T3SA basal body and for the surface localization of PcrV and PopB.

It has been shown that some PcrV-based vaccines and antibodies can block Pa cytotoxicity (39, 40). The ability to inhibit cytotoxicity that has been associated with anti-PcrV antibodies is presumably caused by antibody binding to the T3SA needle tip to neutralize secretion. Therefore, the ability to prevent cytotoxicity by sera from mice vaccinated with L-PaF was assessed (**Figure 3****, bottom**). As expected, sera from PBS vaccinated mice did not reduce killing of the A549 cells while sera from PcrV+dmLT vaccinated mice significantly reduced the observed cytotoxicity. The cytotoxicity was also reduced by the sera from mice vaccinated with 20 µg and 10 µg L-PaF as compared to the Pa treated sample. Additionally, Like the OPK activity, the inhibition of cytotoxicity by L-PaF was dose dependent. The specificity and dose dependence of the OPK activity and inhibition of cytotoxicity in sera from mice vaccinated with L-PaF are key findings since these activities are considered important for a successful Pa vaccine candidate

### L-PaF Promotes Bacterial Clearance From the Lungs of Vaccinated Mice

**Figure 4:**
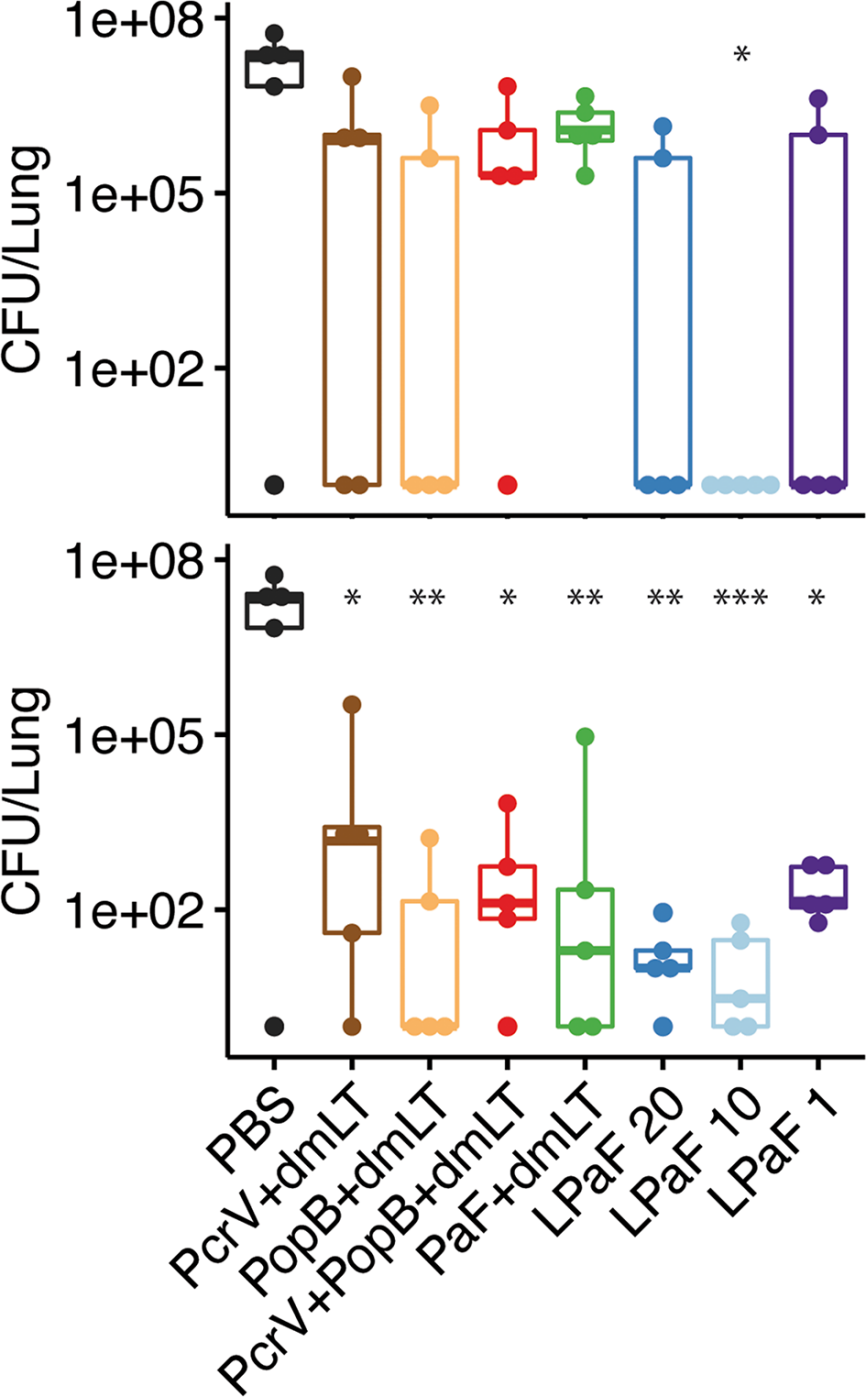
Protective efficacy of antigen formulations against *Pseudomonas aeruginosa* (Pa) challenge in mice. BALB/c mice (n=10) were vaccinated intranasally (IN) on days 0, 14, and 28 with PcrV (10 µg), PopB (2.5 µg), PcrV and PopB (10 µg+2.5 µg), or PaF (20 µg) admixed with dmLT (2.5 µg) or they were vaccinated with 20, 10, or 1 µg of LTA1-GSAAS-PaF (L-PaF). On day 56, the mice were challenged IN with mPA08-31. On each of days 3 (top panel) and 4 (bottom panel), the CFU/lung was determined (n=5). 10^0^ is used to denote mice that cleared the infection (had no detectable CFU). It should be noted that all of the PBS vaccinated mice died between days 3 and 4. Thus, no significance could be assigned using those lung burden results. Because of this, the CFU from the PBS vaccinated mice from day 3 was used to determine day 4 significance. Significance was calculated by comparing antigen containing groups to the PBS group with Dunnett’s multiple comparisons tests. *p < 0.05; **p < 0.01; ***p < 0.001.

Having established that an immune response had been elicited by L-PaF, these mice were challenged on day 56 with 2x10^7^ mPA08-31. On day 3 post-infection the lungs from five mice were harvested for assessment of CFU burden and the post-challenge immune response. When compared to PBS immunized mice, the bacterial burden in the majority of the lungs examined for all the protein antigen vaccinated groups was substantially reduced (**Figure 4****, top**). While there is no significance between the antigen-vaccinated groups, there is a trend that parallels what is seen with OPK activity. PcrV, PopB, PcrV+PopB, all admixed with dmLT, and the 1 µg L-PaF have lower levels of Pa clearance than the other L-PaF groups and the PopB+dmLT vaccinated group. Indeed, mice that were vaccinated with 10 µg L-PaF all completely cleared the pathogen. When a second set of five mice were checked for bacterial lung burden on day 4 post-infection, all of the PBS vaccinated mice had died while all of the vaccinated mice survived with lung burdens reduced even further than what was seen on day 3 (**Figure 4****, bottom**). Because the PBS vaccinated mice had all succumbed to the infection by day 4, statistical analysis to compare the CFU burden in PBs mice verses the other vaccinated mice was not possible. However, if the CFU levels of the PBS-vaccinated mice from day 3 were used in the statistical analysis, all antigen-vaccinated mice were statistically different from the PBS-vaccinated mice. These results are consistent with day 3 with the 10 µg L-PaF and PopB+dmLT vaccinated mice having the largest number of mice completely clearing the pathogen. Thus, the L-PaF vaccine candidate is able to promote bacterial clearance from mice challenged with Pa.

### L-PaF Induces Expression of IL-17A

**Figure 5:**
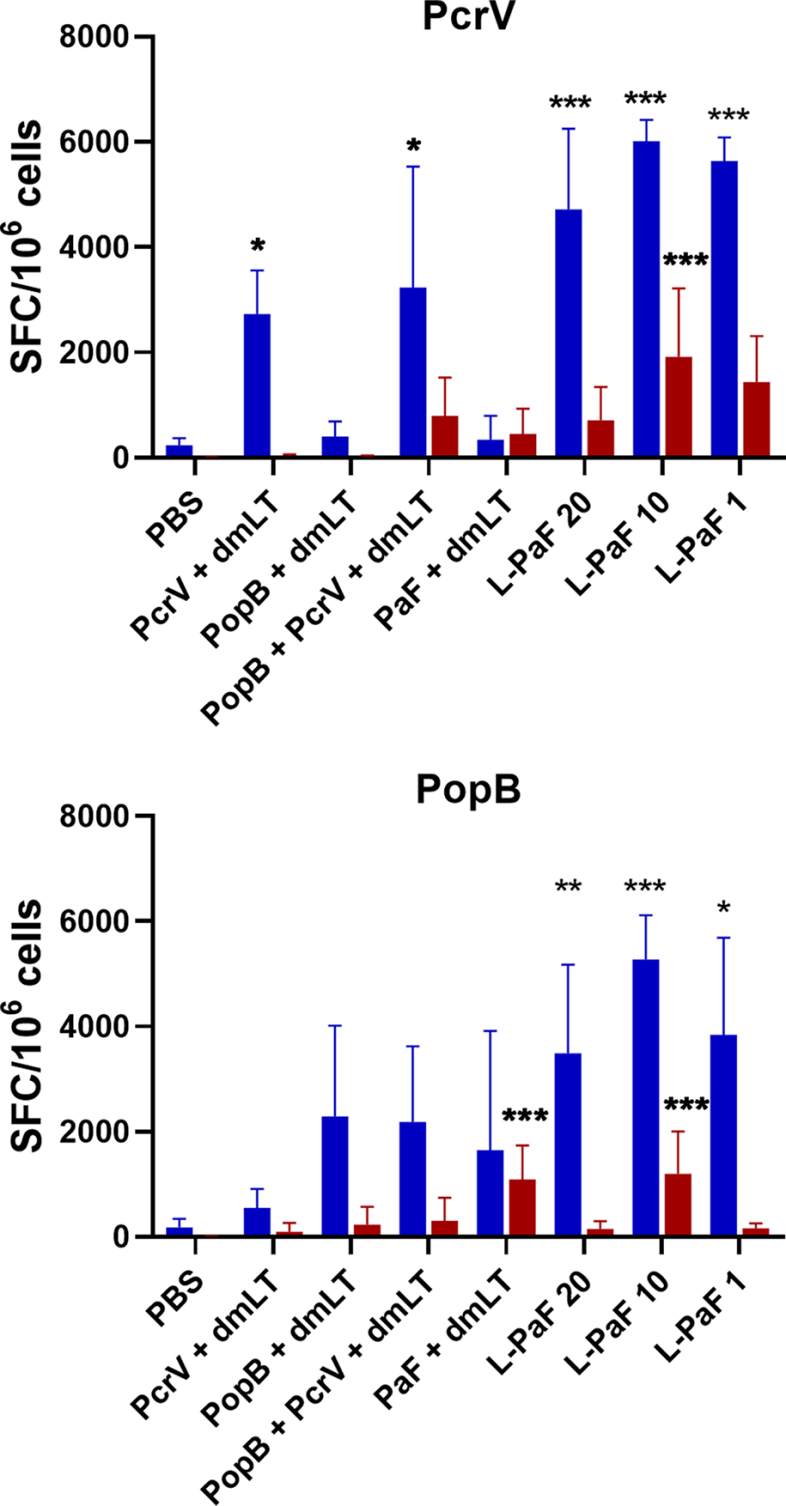
Frequency of IL-17 and IFN-γ secreting cells after antigen-specific stimulation. The single lung cell suspensions from **Figure 4** were used to assess antigen specific IL-17 and IFN-γ secreting cells. Cells were incubated with 10 µg PcrV (top) or PopB (bottom). IL-17 (blue bars) and IFN-γ (red bars) secreting cells were enumerated by ELISpot and are presented here as spot forming cells/10^6^ cells. The data are plotted as means ± SD for individual mice in each group. Significance was calculated by comparing groups that were unvaccinated (PBS) and mice vaccinated with antigens using two-way ANOVA. *p < 0.05; **p < 0.01; ***p < 0.001.

The lungs from the mice used to determine CFU burden were also examined for post-challenge cellular immune responses. The single cell suspensions were stimulated with PcrV or PopB and the number of cells secreting IL-17A and IFN-γ cells was quantified by ELISpot analysis (**Figure 5**). Unstimulated cells served as a negative control. Regardless of stimulation with PcrV (**Figure 5****, top, blue bars**) or PopB (**Figure 5****, bottom, blue bars**), lung cells from all L-PaF vaccinated groups had a significantly higher frequency of IL-17A secreting cells compared to PBS vaccinated controls. In contrast, only the PcrV stimulated cells from groups vaccinated with PcrV or PcrV+PopB admixed with dmLT had significantly higher frequencies of IL-17A secreting cells with no significantly higher frequencies detected in the other groups after PcrV or PopB stimulation. Significantly higher frequencies of IFN-γ secreting cells was observed only in the lung for the 10 µg L-PaF immunized group stimulated with PcrV (**Figure 5****, top, red bars**) or PopB (**Figure 5****, bottom, red bars**) and in the PaF+dmLT vaccinated group after stimulation with PopB.

**Figure 6:**
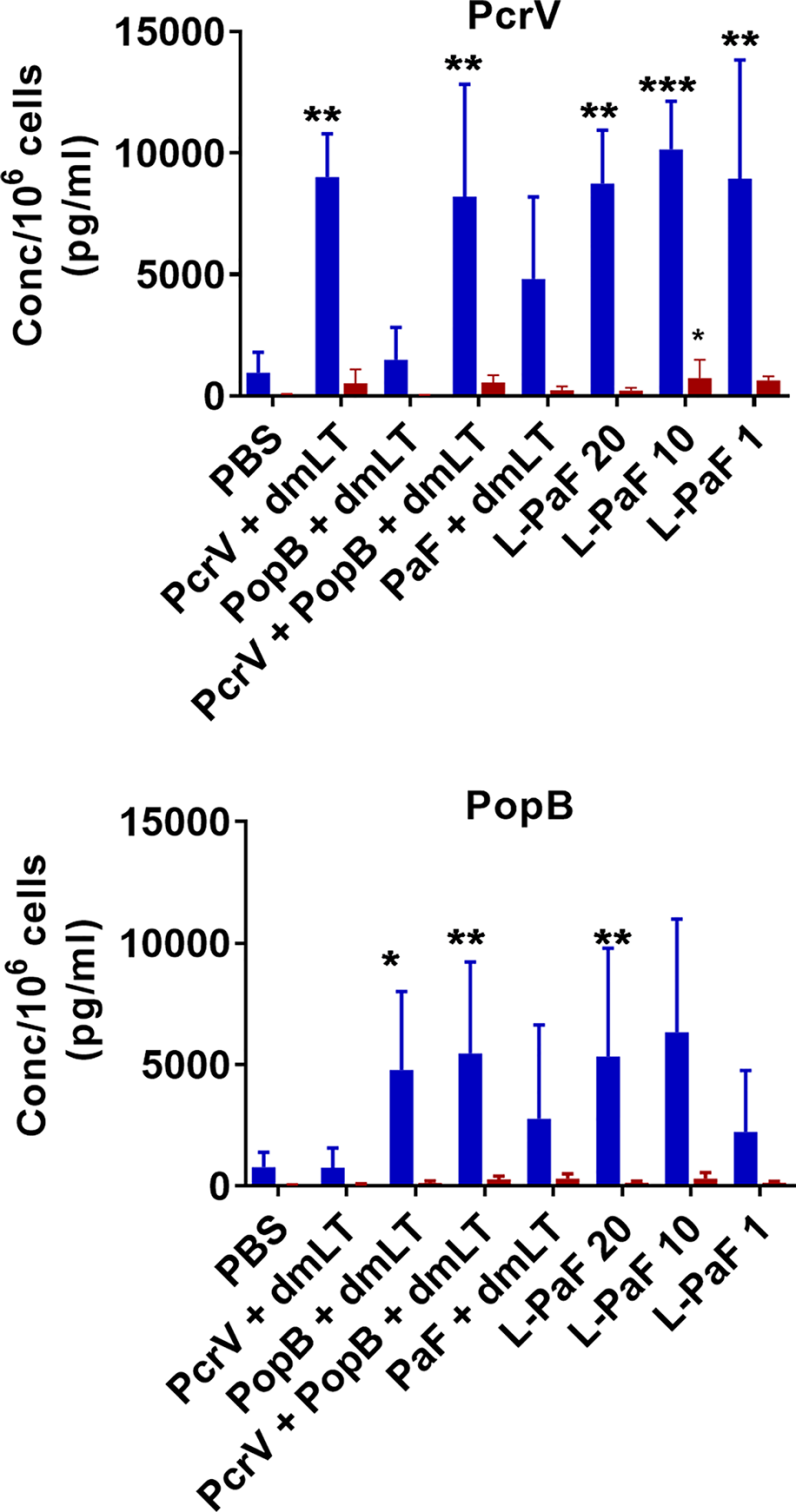
Levels of IL-17 and IFN-γ secreted from lung cells after stimulation with PcrV or PopB. The single lung cell suspensions from **Figure 4** were used to assess antigen specific IL-17 and IFN-γ secretion from lung cell suspensions. Cells were incubated with 10 µg PcrV (top) or PopB (bottom). IL-17 (blue bars) and IFN- γ (red bars) levels were determined by MesoScale Discovery analysis as per manufacturer’s specifications and are presented here as pg/ml/10^6^ cells. The data are plotted as means ± SD for individual mice in each group. Significance was calculated by comparing groups that were unvaccinated (PBS) and mice vaccinated with antigens using two-way ANOVA. *p < 0.05; **p < 0.01; ***p < 0.001.

Cytokine levels in the supernatant fractions of lung cells were also evaluated after stimulation with PcrV (**Figure 6****, top, blue bars**) and PopB (**Figure 6****, bottom, blue bars**). When compared to the PBS vaccinated group, significantly higher levels of IL-17A were secreted from cells in all the L-PaF groups after stimulation with PcrV. This was also seen in the PcrV+dmLT and PcrV+PopB+dmLT vaccinated groups. In contrast, only the 20 µg L-PaF, PcrV+PopB+dmLT and PopB+dmLT vaccinated groups elicited significantly higher IL-17A secretion after PopB stimulation. Only the 10 µg L-PaF group secreted significantly higher IFN-γ as compared to PBS groups when stimulated with PcrV though the levels were still modest (**Figure 6****, top, red bars**). While other cytokines were examined, no noteworthy trends were observed. The increase in IL-17A secreting cells, as well as its secretion by lung cells from mice vaccinated with L-PaF, is important because as with OPK activity it is postulated that a successful vaccine candidate will need to induce an IL-17 response to clear Pa. While there are some slight differences in the responses for each group with regard to increased frequency of the IL-17A and IFN-γ secreting cells and the levels of secreted IL-17A and IFN-γ, the trends for the two cytokines are similar with the L-PaF eliciting a greater IL-17A response. Taken together with the OPK and protection, the L-PaF appears to be a better vaccine candidate than using a single protein or a combination of proteins with a separate dmLT adjuvant.

### Assessment of L-PaF Induced Frequency of IL-17A and IFN-γ Secreting Cells and IL-17A and IFN-γ Levels Pre-Challenge

**Figure 7:**
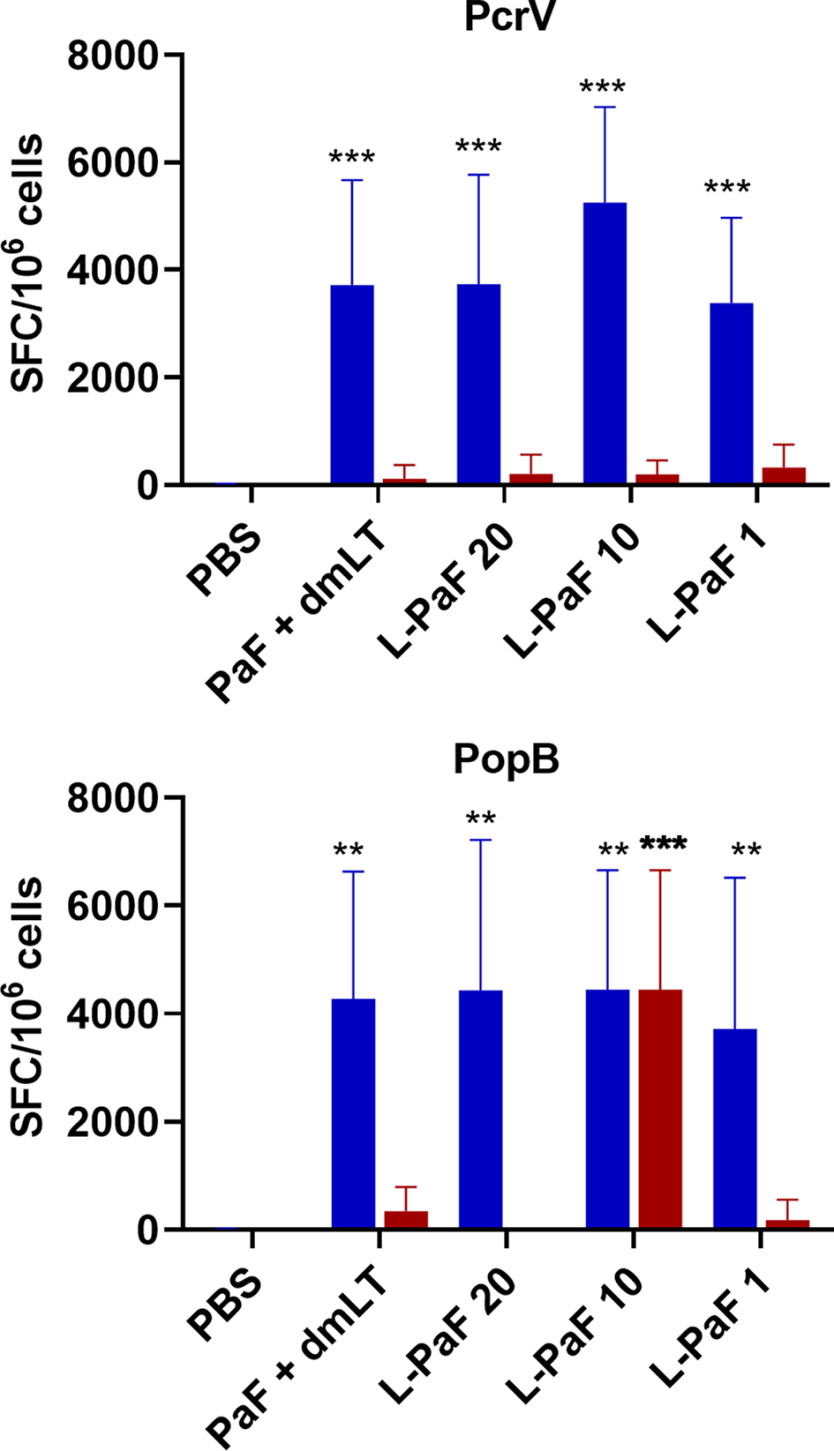
Frequency of IL-17 and IFN-γ secreting cells from pre-challenge mice after antigen-specific stimulation. Mice (n=10) vaccinated with PBS, PaF (20 µg) admixed with dmLT (2.5 µg) or with 20, 10 or 1 µg of LTA1-GSAAS-PaF (L-PaF) on days 0, 14, 28. The single lung cell suspensions were obtained and used to assess antigen specific IL-17 and IFN-γ secreting cells. Cells were incubated with 10 µg PcrV (top) or PopB (bottom). IL-17 (blue bars) and IFN-γ (red bars) secreting cells were enumerated by ELISpot and are presented here as spot forming cells/10^6^ cells. The data are plotted as means ± SD for individual mice in each group. Significance was calculated by comparing groups that were unvaccinated (PBS) and mice vaccinated with antigens using two-way ANOVA. **p < 0.01; ***p < 0.001.

**Figure 8:**
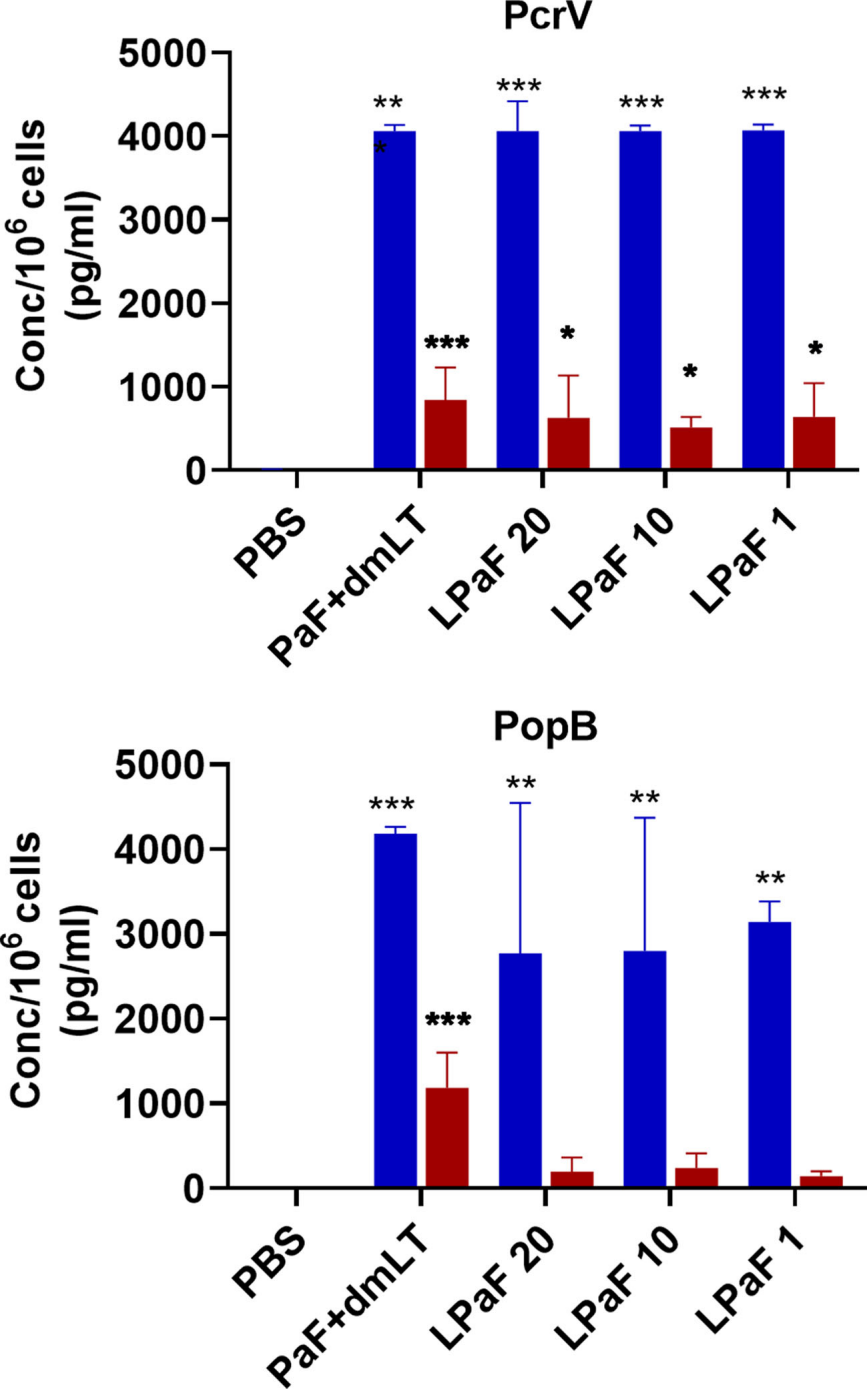
Levels of IL-17 and IFN-γ secreted from pre-challenge mice lung cells after stimulation with PcrV or PopB. The single lung cell suspensions from **Figure 7** were used to assess antigen specific IL-17 and IFN-γ secretion from lung cell suspensions. Cells were incubated with 10 µg PcrV (top) or PopB (bottom). IL-17 (blue bars) and IFN-γ (red bars) levels were determined by MesoScale Discovery analysis as per manufacturer’s specifications and are presented here as pg/ml/10^6^ cells. The data are plotted as means ± SD for individual mice in each group. Significance was calculated by comparing groups that were unvaccinated (PBS) and mice vaccinated with antigens using two-way ANOVA. *p < 0.05; **p < 0.01; ***p < 0.001.

Because, when taken together, L-PaF was deemed to have elicited a better protective immune response in mice than the other antigens+dmLT, PcrV, PopB alone or together were no longer examined. A subsequent set of mice (n=15/group) was vaccinated IN three times on days 1, 14, 28 with PBS, PaF+dmLT, and 1, 10, or 20 µg of L-PaF. On day 55, five mice were euthanized and the pre-challenge IL-17A and IFN-γ responses in lung cells were examined (**Figures 7** and **8**). The frequencies of IL-17A secreting cells after stimulation with either PcrV (**Figure 7****, top**, **blue bars**) or PopB (**Figure 7****, bottom, blue bars**) were significantly increased when compared to the PBS vaccinated mice, regardless of antigen. In contrast, the frequency of IFN-γ secreting cells was increased significantly in the 10 µg L-PaF vaccinated group, but only after stimulation with PopB (**Figure 7****, bottom, red bars**). A similar pattern was seen when cytokine secretion from the lung cells (**Figure 8**). Regardless of the stimulating antigen, a significant increase in IL-17A was seen from lung cells in all four antigen vaccinated groups.

### L-PaF Induces a Strain-Independent Protective Immune Response

**Figure 9:**
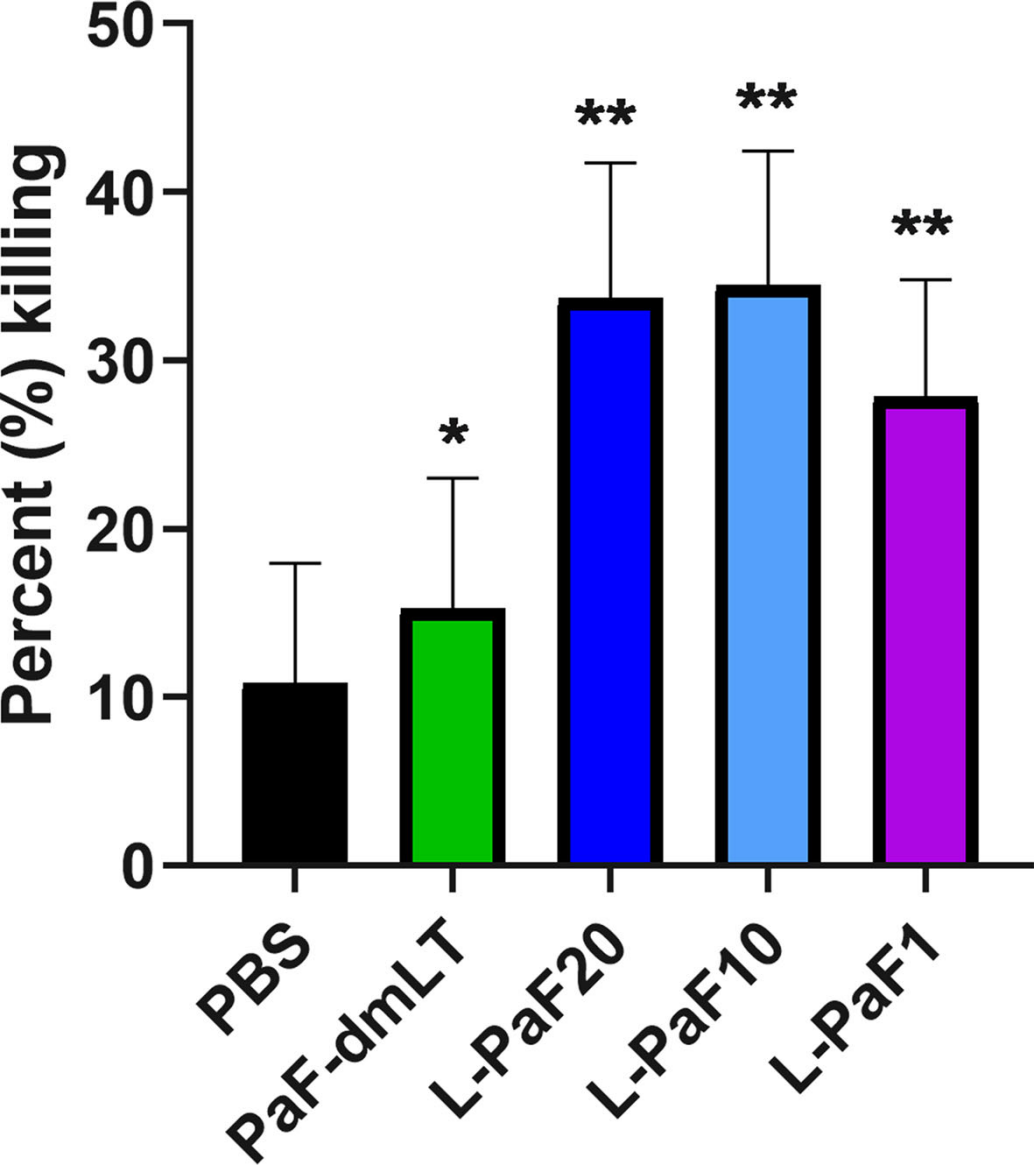
Opsonophagocytic killing (OPK) activity of serum samples. Pooled sera from each vaccinated mouse group (5 µl/mouse) were tested for their OPK activity after heat-inactivation. The sera were combined with mPA08-31 and J774A.1 cells at an MOI of 0.1. Killing was determined using (T_0_-T_30_)/T_0_x100. This is a representative experiment of quadruplicates for each sample. **p < 0.005 and *p < 0.01 for respective groups were compared to PBS using a two-way ANOVA.

**Figure 10:**
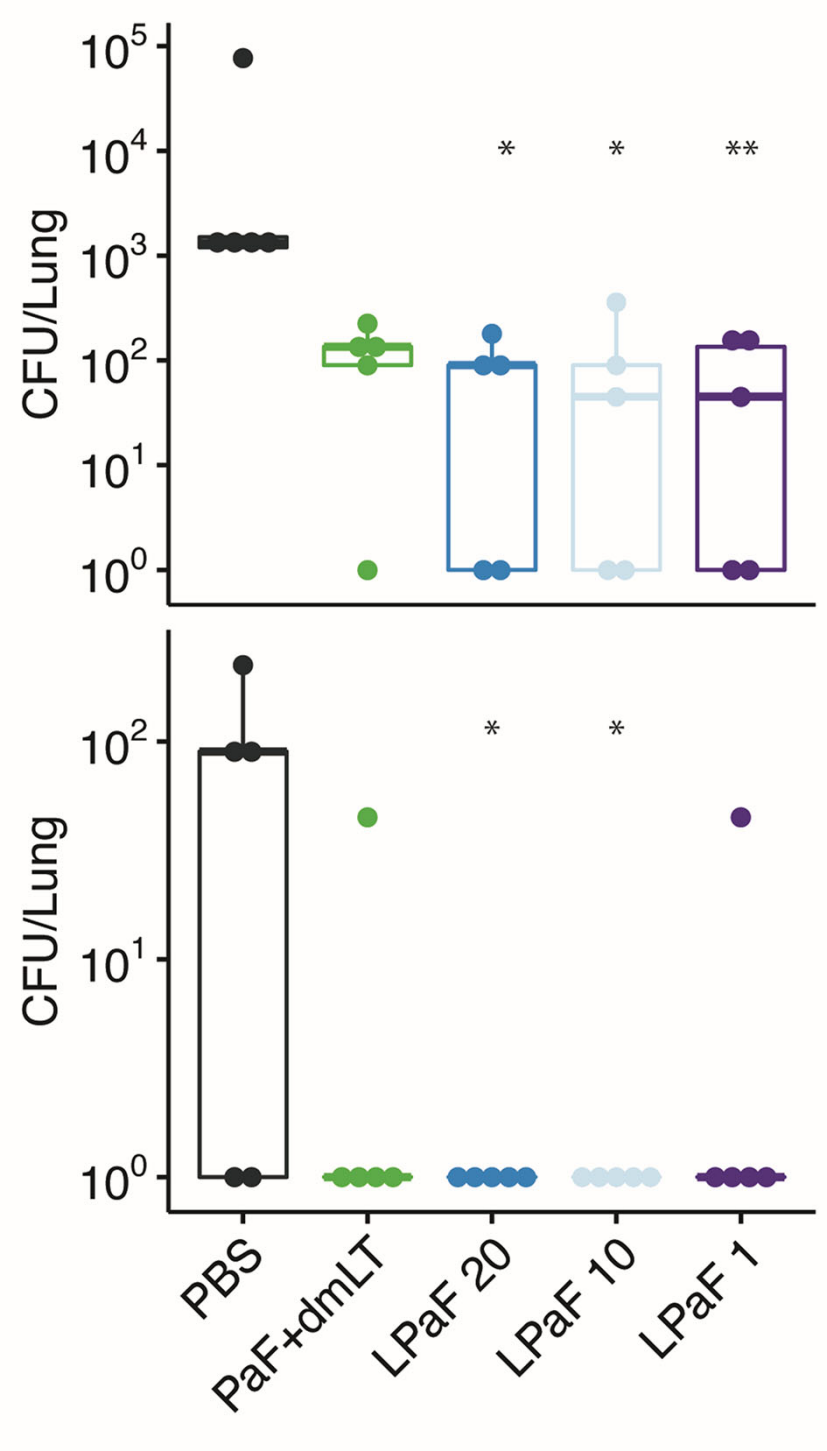
Protective efficacy of antigen formulations against a second Pa challenge strain in mice. Mice (n=10) vaccinated with PBS, PaF (20 µg) admixed with dmLT (2.5 µg) or with 20, 10 or 1 µg of L-PaF on days 0, 14, 28. On day 56, the mice were then challenged IN with PA15808959 and CFU/lung determined on days 2 (top) and 3 (bottom) (n=5 on each day). The 10^-1^ and 10^0^ designations are the same as in **Figure 4**. In all cases, the black line indicates the mean counts for the group. Significance was calculated by comparing antigen containing groups to the PBS group with Dunnett’s multiple comparisons tests. *p < 0.05; **p < 0.01.

Sera from another group of mice vaccinated with PaF+dmLT or L-PaF were assessed for anti-PcrV and anti-PopB IgG and IgA titers with the results being the same as those shown in **Figure 2** (data not shown). These serum samples were then used to determine the OPK activity against a different Pa strain (**Figure 9**). As above, sera from the L-PaF groups killed 27-24% of PA15808959 while the PaF+dmLT only elicited 15% killing activity. The remaining 10 mice from each group were then challenged with 1x10^5^ CFU of PA15808959. Since PA15808959 is a more virulent Pa strain, a lower challenge dose was used and the mice were sacrificed at days 2 and 3 post-infection (**Figure 10**). When compared to PBS, the bacterial burden in the lungs was reduced significantly at day 2 for the groups vaccinated with L-PaF (**Figure 10****, top**). When the day 3 post-infection burdens were assessed, the 10 and 20 µg L-PaF immunized mice had no detectable Pa in the lungs and the PaF+dmLT and 1 µg L-PaF immunized mice had no detectable burden in all but one mouse (**Figure 10****, bottom**). In contrast, while two of the PBS vaccinated mice did clear the Pa, the other three mice had overall higher burdens.

**Figure 11:**
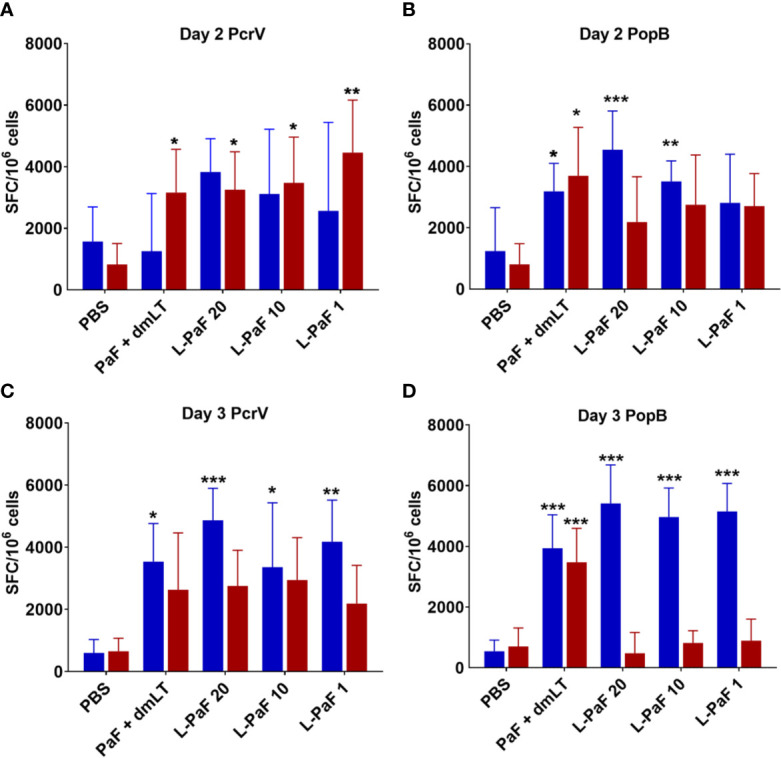
Frequency of IL-17 and IFN-γ secreting cells from mice after antigen-specific stimulation. Mice (n=10) vaccinated with PBS, PaF (20 µg) admixed with dmLT (2.5 µg) or with 20, 10 or 1 µg of L-PaF on days 0, 14, 28. The single lung cell suspensions were obtained and used to assess antigen specific IL-17 and IFN-γ secreting cells. Cells were incubated with 10 µg PcrV (top) or PopB (bottom). IL-17 (blue bars) and IFN-γ (red bars) secreting cells were enumerated by ELISpot and are presented here as spot forming cells/10^6^ cells. The data are plotted as means ± SD for individual mice in each group. Significance was calculated by comparing groups that were unvaccinated (PBS) and mice vaccinated with antigens using two-way ANOVA. *p < 0.05; **p < 0.01; ***p < 0.001.

**Figure 12:**
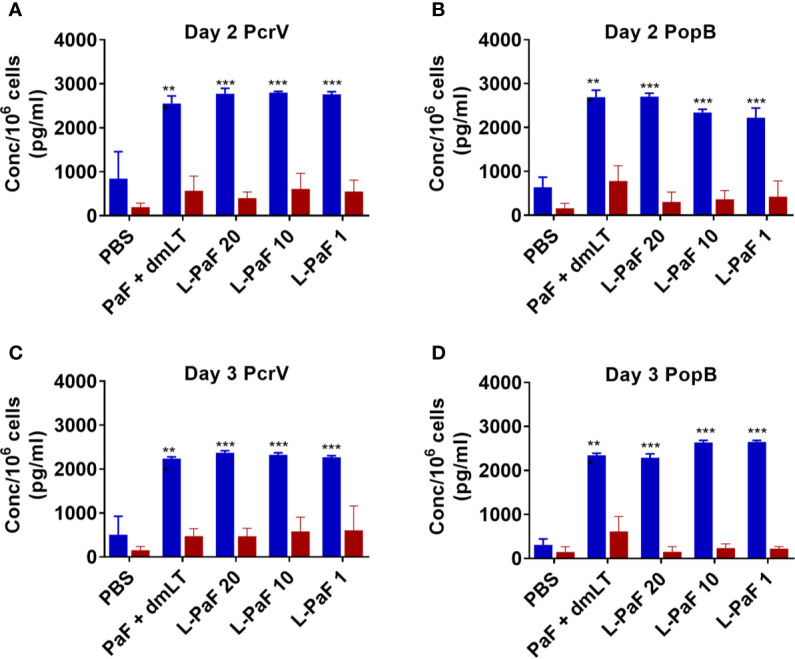
Levels of IL-17 and IFN-γ secreted from lung cells after stimulation with PcrV or PopB. The single lung cell suspensions from **Figure 7** were used to assess antigen specific IL-17 and IFN-γ secretion from lung cell suspensions. Cells were incubated with 10 µg PcrV (top) or PopB (bottom). IL-17 (blue bars) and IFN-γ (red bars) levels were determined by MesoScale Discovery analysis as per manufacturer’s specifications and are presented here as pg/ml/10^6^ cells. The data are plotted as means ± SD for individual mice in each group. Significance was calculated by comparing groups that were unvaccinated (PBS) and mice vaccinated with antigens using two-way ANOVA. **p < 0.01; ***p < 0.001.

Cells from the lung used for determining bacterial burdens were also used to assess antigen-specific IL-17A and IFN-γ (**Figure 11**) as well as the levels of cytokine secretion from the cells after antigen stimulation (**Figure 12**). On day 2, the frequency of IL-17A secreting lung cells was significant in the PaF+dmLT, 10 and 20 µg L-PaF vaccinated groups after stimulation with PopB (**Figure 11B**). While other groups had elevated frequencies, none were statistically significant (**Figures 11A, B**). In contrast, the frequencies of IFN-γ secreting lung cells were significant in all antigen groups after stimulation with PcrV and in the PaF+dmLT group stimulated with PopB (**Figures 11A, B**). On day 3, all groups from antigen vaccinated mice exhibited significant frequencies of antigen-specific IL-17A cells while only while only the group from PaF+dmLT mice showed significant IFN-γ secreting cells after stimulation with PopB (**Figures 11C, D**). When the levels of secreted IL-17A and IFN-γ were examined following antigen stimulation, all groups vaccinated with a protein antigen, regardless of day post-infection or antigen stimulation, secreted significantly higher levels of IL-17A into the supernatant (**Figure 12**). In contrast, none of the groups from either day or after stimulation with PcrV or PopB secreted significant levels of IFN-γ (**Figure 12**). With the exception of the PaF+dmLT group, these levels are consistent with the IL-17A and IFN-γ results seen after challenge with mPA08-31 (**Figs. 5 and 6**) and the pre-challenge IL-17A and IFN-γ results (**Figs. 7 and 8**). IL-17A has been previously reported to be negatively correlated with lung bacterial burden (17), but while there is no reported role of IFN-γ in resolution of *Pseudomonas* infection, it was included in the current study because some of the groups showed significant induction of this cytokine. The potential role of IFN-γ will be further examined in future studies. From the current experiments, it is clear that the L-PaF alone can elicit an immune response with high antibody titers that can elicit OPK. This likely contributed to bacterial clearing in the lung and this may be complemented by antibody neutralization of the T3SS. Similarly, L-PaF elicited high levels of IL-17A, which again functions to clear the bacteria *via* a Th17 cellular response.

The L-PaF subunit vaccine delivered IN elicits strong IgG, IgA and IL-17A responses. A comparison of pre-challenge immunological markers with lung CFUs from challenged mice suggests that lung IL-17 is important for host protection (**Table 1**). Five of the 10 factors most strongly correlated to post-challenge lung CFUs are IL-17-related, including the most strongly correlated factor, PcrV lung IL-17 (Pearson r = -0.995). IL-1β, which has been shown to be required for an IL-17 response (41), may also strongly correlate to protection though those responses were not included here. These findings are in agreement with previous studies reporting that IL-17 is crucial for protection against Pa infection (42).

**Table 1:** Ten pre-challenge immune markers most strongly correlated with post-challenge lung CFUs.

**Immune marker**	**Pearson’s r**
PcrV Lung IL-17A	−0.995
PcrV Lung IL-1β	−0.871
PopB Lung IL-17A	−0.815
PcrV Lung TNF-α	−0.749
PcrV Lung IL-17A Elispot	−0.659
PopB Lung IL-17A Elispot	−0.644
PcrV Lung IFN-γ	−0.643
PcrV Spleen IL-17A	−0.571
PcrV Lung IL-6	−0.551
PopB Lung IL-12p70	−0.547

Ten largest Pearson’s r coefficients between immune markers in individual pre-challenge mice and group-average lung CFUs on day 2 post-challenge. The 10 largest Pearson’s r coefficients were determined to allow assessment of the correlation between immune markers in the individual pre-challenge mice in **Figures 7** and **8** (or cytokines not shown here) and the group-average lung CFUs on day 2 post-challenge of **Figure 10**, top panel.

### L-PaF Vaccination Enhances Bacterial Clearance of Pa From the Lungs of Rats

**Figure 13:**
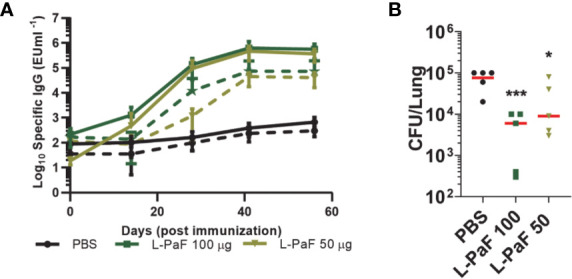
Kinetics of IgG/IgA titers and protective efficacy of LTA1-GSAAS-PaF (L-PaF) in rats. Rats were vaccinated three time by the IN route (Days 0, 14 and 28). Blood samples were collected and serum was separated. **(A)** IgG (solid lines)/IgA (broken lines) antibodies specific for PcrV or PopB were measured by ELISA. The individual titers are represented as EU ml^-1^, and each point represents mean ± S.D. of 10 mice per group. Statistical significance was calculated by comparing L-PaF 100 and 50 to PBS groups using a one-way ANOVA. **(B)** These rats were challenged IN on day 56 for comparison with PBS vaccinated mice. Statistical significance was calculated by comparing L-PaF 100 µg or L-PaF 50 µg to PBS immunized group using a one-way ANOVA. *p < 0.05; ***p < 0.001.

Rats were vaccinated at Days 0, 14, and 28 with 50 μg or100 μg L-PaF with PBS being used as the negative control. Blood was collected at each vaccination date, as well as on days 42 and 56 for immunologic analysis. The kinetics of anti-PcrV and anti-PopB IgG titers were assessed by ELISA (**Figure 13A**). The day-42 post-vaccination sera from L-100 µg PaF and 50 µg L-PaF vaccinated groups showed considerable OPK activity with 54% and 50%, respectively. Differences in the OPK activities between these two groups were statistically insignificant (p >0.05).

The rats (n=5) were challenged with 1 x 10^7^ CFUs of mPA08-31 and euthanized at day 3 post-infection to assess bacterial burden in the lung (**Figure 13B**). Compared to the PBS control, the 50 µg L-PaF vaccinated group showed a reduction in lung burden to 2.7 x 10^4^ (p<0.05). The 100 µg L-PaF group had an even more significant reduction in lung burden to 2 x 10^3^, almost two-logs lower than the PBS control (p<0.001). These results indicate that L-PaF can elicit efficient protection against Pa in an alternative animal model.

## Conclusions

We demonstrate here that a novel subunit vaccine comprised of a recombinant fusion protein can elicit a protective immune response that protects both mice and rats against infection by *P. aeruginosa*. A feature of this vaccine is that it contains two antigens from the Pa T3SS that have previously been shown to offer at least some protection on their own against Pa challenge and a protein that imparts self-adjuvanticity to the fusion protein. The use of PcrV and PopB is expected to provide an immune response that is largely independent of serotype and protects against multiple Pa strains. The resulting immune response to generates antibodies that possess opsonophagocytic killing activity and are able to inhibit Pa cytotoxicity (usually attributed to anti-PcrV antibodies), which are important hallmarks of a protective Pa vaccine. Additionally, LTA1 provides adjuvant activity to the vaccine and ensures that adjuvant and antigen are taken up by the same antigen-presenting cell. Intranasal (IN) vaccinations using dmLT have been shown to elicit IL-17A responses (23) and this is proposed to be important for eliciting immunological protection against the establishment of Pa infection (4).

While L-PaF will provide protection against those Pa strains of the PA01/PA014 clades, it will not provide protection against those strains of the PA7-like clade that lack the T3SS. These strains kill cells *via* exolysin A (ExlA) rather than the T3SS (43). It has been proposed that high concentrations of ExlA pool at the interface of the type IV pili:host cell surface, which promotes host cell lysis (44). However, ExlA from Pa or produced recombinantly cannot lyse eukaryotic cells (43). Generation of a vaccine against this outlier group will require special considerations that will be addressed in future studies. Nevertheless, the results presented here show that the L-PaF vaccine promotes protective immunity against two different Pa clinical isolates and is effective in both mouse and rat models. Furthermore, this protective vaccine requires purification of only a single fusion protein/adjuvant and is anticipated to be safer for IN administration than an antigen admixed with dmLT (22). Additional optimization of this vaccine platform through the testing of multiple formulations is under way and should provide additional benefits with regard to further improved efficacy and yield information regarding the mechanisms that govern the efficacy of L-PaF.

## Data Availability Statement

The raw data supporting the conclusions of this article will be made available by the authors, without undue reservation.

## Ethics Statements

The mouse studies were reviewed and approved by University of Kansas Institutional Animal Care and Use Committee. The rat animal protocols were reviewed and approved by the University of Alabama at Birmingham Institutional Animal Care and Use Committee Practices.

## Author Contributions

WLP,WDP and SB conceptualized the study. WLP, WDP, SD, QZ and SB designed the study. SD, DH, QZ, SR, TL, JK, SB, and WDP performed experiments. SD, QZ, TL, WDP, WLP and SB analyzed the data. TL, SW and SB performed statistical analysis. WLP wrote first draft of the manuscript with inputs from all authors. WDP and SW revised the manuscript. All authors contributed to the article and approved the submitted version.

## Funding

This work was funded by NIAID grants R01AI138970 and R21AI140701 to WLP.

## Conflict of Interest

SW and WP were employed by Hafion LLC.

The remaining authors declare that the research was conducted in the absence of any commercial or financial relationships that could be construed as a potential conflict of interest.
