# Supplementary material for: Development of a Broadly Protective, Self-Adjuvanting Subunit Vaccine to Prevent Infections by Pseudomonas aeruginosa
Source: Front Immunol. 2020 Nov 17;11:583008. doi: 10.3389/fimmu.2020.583008 (PMC7705240; doi:10.3389/fimmu.2020.583008)
Supplement: Supplementary file 1 [file DataSheet_1.docx]

**Supplemental Information**

**Development of a broadly protective, self-adjuvanting subunit vaccine to prevent infections by *Pseudomonas aeruginosa***

Sayan Das^1¶^, Debaki R. Howlader^1¶^, Qi Zheng^1¶^, R. Siva Sai Kumar^1^, Sean K. Whittier^1,2^, Ti Lu^1^, Johnathan D. Keith^3^, William D. Picking^1^, Susan E. Birket^3^ and Wendy L. Picking^1,2*^

^1^Department of Pharmaceutical Chemistry, School of Pharmacy, University of Kansas, Lawrence, KS, ^2^Hafion, LLC, Lawrence, KS, and ^3^Department of Medicine and Gregory Fleming James Cystic Fibrosis Research Center, School of Medicine, University of Alabama at Birmingham, Birmingham, AL

^¶^ These authors contributed equally to this work.

^*^ To whom correspondence should be directed: Email: [wendy.picking@ku.edu](mailto:wendy.picking@ku.edu)

Keywords: *Pseudomonas aeruginosa*, Type III secretion system, vaccine, IL-17, opsonophagocytosis, protective efficacy, PcrV, PopB.

**Acronyms used in this manuscript:**

| ADPr | ADP-ribosylation |
| --- | --- |
| AEBSF | 4-benzenesulfonyl fluoride hydrochloride, protease inhibitor |
| ARF4 | ADP-ribosylation factor 4 |
| BMDC | Bone marrow dendritic cell |
| CDC | Center for Disease Control |
| DBF | IpaD-IpaB fusion |
| dmLT | Double mutant labile toxin from Enterotoxigenic *E. coli* |
| FF | Fast Flow |
| GM-CSF | granulocyte-macrophage colony-stimulating factor |
| HT | His-tag |
| HT-PcrH | HT-PcrH, the PopB chaperone |
| IN | Intranasal |
| IMAC | immobilized metal affinity chromatography |
| IpaD | *Shigella* spp T3SA tip protein |
| IpaB | *Shigella* spp T3SA first translocator protein |
| LDAO | Lauryldimethylamine oxide |
| L-DBF | The fusion of L-IpaD-IpaB |
| L-PaF | The fusion of LTA1-PcrV-PopB |
| LTA1 | A1 subunit from dmLT or LT |
| MDR | Multi-drug resistant |
| OMV | Outer membrane vesicles |
| OPK | Opsonophagocytic killing |
| Pa | *Pseudomonas aeruginosa* |
| PaF | PaFusion – fusion of PcrV and PopB |
| PcrV | Pa T3SA tip protein |
| PopB | Pa T3SA first translocator protein |
| Q column | Q FF anion exchange chromatography |
| TB | Terrific Broth |
| T3SA | Type III secretion system apparatus |
| T3SS | Type III secretion system |
| WCK | Whole cell, killed |

**Amino acid sequence of L-PaF:**

LTA1 sequence is in black. PcrV sequence is in green. Linker sequence is GS. PopB sequence is in Orange

MDNGDRLYRADSRPPDEIKRSGGLMPRGHNEYFDRGTQMNINLYDHARGTQTGFVRYDDGYVSTSLSLRSAHLAGQSILSGYSTYYIYVIATAPNMFNVNDVLGVYSPHPYEQEVSALGGIPYSQIYGWYRVNFGVIDERLHRNREYRDRYYRNLNIAPAEDGYRLAGFPPDHQAWREEPWIHHAPQGCGNSSRMEVRNLNAARELFLDELLAASAAPASAEQEELLALLRSERIVLAHAGQPLSEAQVLKALAWLLAANPSAPPGQGLEVLREVLQARRQPGAQWDLREFLVSAYFSLHGRLDEDVIGVYKDVLQTQDGKRKALLDELKALTAELKVYSVIQSQINAALSARQGIRIDAGGIDLVDPTLYGYAVGDPRWKDSPEYALLSNLDTFSGKLSIKDFLSGSPKQSGELKGLSDEYPFEKDNNPVGNFATTVSDRSRPLNDKVNEKTTLLNDTSSRYNSAVEALNRFIQKYDSVLSDILSAIGSMNPITLERAGLPYGVADAGDIPALGRPVARDVESLRVERLAAPAAASASGTGVALTPPSAASQQRLEVANRAEIASLVQAVGEDVGLARQVVLAGASTLLSAGLMSPQAFEIELAKITGEVENQQKKLKLTEIEQARKQNLQKMEDNQQKIRESEEAAKEAQKSGLAAKIFGWISAIASIIVGAIMVATGVGAAAGALMIAGGVMGVVSQSVQQAAADGLISKEVMEKLGPALMGIEMAVALLAAVVSFGGSAVGGLARLGAKIGGKAAEMTASLASKVADLGGKFGSLAGQSLSHSLKLGVQVSDLTLDVANGAAQATHSGFQAKAANRQADVQESRADLTTLQGVIERLKEELSRMLEAFQEIMERIFAMLQAKGETLHNLSSRPAAI-.

**
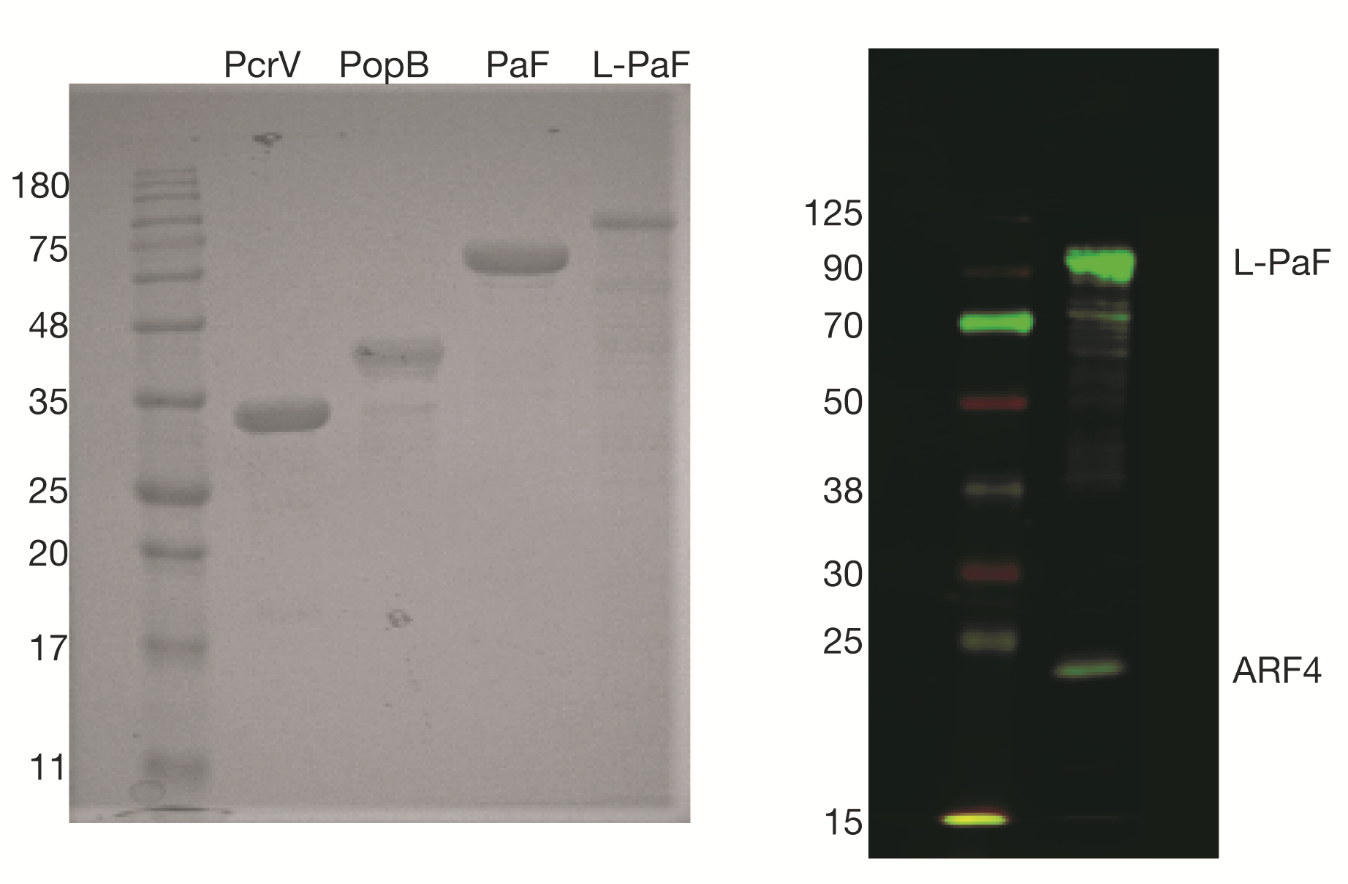
**

**Supplemental Figure S1. Preparation of the recombinant proteins used in this study.** A) Four recombinant proteins were tested in this study. PcrV (tip protein) and PopB (first translocator protein) from the *Pseudomonas aeruginosa* T3SS were purified as described. Then two novel proteins were prepared in which PcrV was fused with PopB (to give PaF) and in which PaF was fused with LTA1 (L-PaF). These four proteins are shown after separation using SDS-PAGE (0.05 µg) and staining with One-Step Blue protein gel stain (Biotium). B) The LTA1 moiety of L-PaF was then shown to transfer biotin-ADPr from biotin-NAD^+^ to itself and ARF4. The biotin-ADPr staining was visualized using western blot analysis followed by probing with Streptavidin–IR800.
